# Patterns of Local Adaptation in the Northern Leopard Frog (*Rana pipiens*) From a Region Undergoing Rapid Climate and Land‐Use Change

**DOI:** 10.1111/eva.70298

**Published:** 2026-07-09

**Authors:** Justin M. Waraniak, David M. Mushet, Craig A. Stockwell

**Affiliations:** ^1^ Department of Biological Sciences, Environmental and Conservation Sciences Program North Dakota State University Fargo North Dakota USA; ^2^ U.S. Geological Survey Northern Prairie Wildlife Research Center Jamestown North Dakota USA

## Abstract

Preservation of local adaptation is a key goal for conservation of populations, and, within the current era of profound environmental change, characterization of local adaptation is necessary to assess the potential resilience of populations to future environmental conditions. In this study, we attempted to characterize patterns of putative, locally adaptive genetic diversity in the northern leopard frog ( *Rana pipiens* ) from an area of the Prairie Pothole Region in North Dakota profoundly impacted by climate and land‐use change. Using a dataset of 25,587 SNPs obtained using RAD‐seq, we characterized population structure, finding similar patterns of isolation by distance and high gene flow as found in previous studies of the region. We considered environmental variables related to climate and several agrochemicals, including nitrates and four commonly applied pesticides, and tested for effects on both neutral and putatively adaptive genetic structure. Genotype by environment analyses identified 388 candidate loci under selection. A large majority of these loci were associated with climatic factors, seemingly mainly driven by differences in breeding‐season precipitation regimes across our study area.

## 1 Introduction

Given the rapid pace of global environmental change, consideration of local adaptation has become an important component in management for the conservation of species and populations. Population genomic approaches have allowed researchers to identify patterns of adaptive diversity that can have important implications for conservation efforts to delineate and manage populations (Forester et al. 2022; Funk et al. 2012). Further, genomic data can provide insights on how populations will respond to future environmental change (Capblancq et al. 2020; DeMarche et al. 2019) and identify candidate populations that will be resilient to current or future environmental conditions (Aitken and Whitlock 2013; Seaborn et al. 2021). Additionally, patterns of local adaptation can reveal aspects of the environment, including anthropogenic stressors, that are imposing selection, and whether populations have the adaptive capacity to respond to environmental changes (Funk et al. 2019; Nicotra et al. 2015).

Rapid environmental change due to land‐use change and climate change has been particularly striking for the Prairie Pothole Region (PPR) in the northern Great Plains of North America, one of the most wetland‐dense regions in the world (Dahl 1990). The PPR is undergoing some of the fastest conversion rates of grassland to cropland, largely due to the growth of corn and soybean farming in the region (Rashford et al. 2011; Wright and Wimberley 2013) which has also resulted in the loss of over half of the wetlands in the PPR (Van Meter and Basu 2015). Further, remaining wetlands may become degraded through the introduction of agrochemicals, including pesticides (Malaj et al. 2020; Williams and Sweetman 2019) and increased nutrient inputs (Martin et al. 2019).

Climate change introduces additional as well as interactive effects with land‐use change that affects PPR wetland ecosystems. Increases in temperature and changes in precipitation regimes can alter hydroperiod and productivity of prairie‐pothole wetlands (Carter Johnson and Poiani 2016; Rashford et al. 2016). Both the timing and magnitude of precipitation events has already caused an unprecedented wet period in the southern PPR (McKenna et al. 2017) and drought in the northwestern PPR (Millett et al. 2009).

Climate shifts and land‐use changes have introduced new selective environments to prairie‐pothole wetlands, as is evidenced by changes in wetland biotic‐community composition (Hu et al. 2022; McLean et al. 2022). Amphibians are among the members of prairie‐pothole wetland biotic communities that may be particularly vulnerable to changes in land‐use and climate. Droughts and increased groundwater withdrawals can shorten hydroperiods in temporary wetlands and lead to local extirpation if amphibian larvae cannot metamorphose before breeding wetlands dry (Corn and Fogleman 1984). Land‐use and climate forces can also increase overwintering mortality in freeze intolerant amphibian species if deepwater, overwintering habitats are too shallow to protect individuals from frost (Mushet 2010). Wetter climates and wetland consolidation due to agricultural drainage can also have negative effects on amphibian populations by reducing the number of small temporary or seasonal wetlands that many amphibians rely on for breeding, and creating larger and more permanent water bodies in which larval amphibians are exposed to more fish predators and other potential competitors (Gabrielsen et al. 2022; Krapu et al. 2018; Wright 2021).

Amphibians are also known to be susceptible to a wide variety of agrochemicals. Nitrate fertilizers can cause both acute and chronic toxicity, delaying metamorphosis or increasing mortality in tadpoles (Hecnar 1995; Orton et al. 2006). Atrazine, one of the most commonly applied agricultural pesticides, is a known endocrine disruptor that affects metabolic energy use, gonadal development, and timing of metamorphosis in larval amphibians (Langlois et al. 2010; Orton et al. 2006; Rohr and McCoy 2010). Atrazine can also lead to immunosuppression in juveniles and adults (Brodkin et al. 2009; Christin et al. 2009). Other common pesticides including glyphosate (Cauble and Wagner 2005; Dinehart et al. 2009), 2,4‐D (Freitas et al. 2022; Pavan et al. 2021), and acetochlor (Crump et al. 2002; Daam et al. 2019) have been associated with detrimental effects on amphibians in controlled experiments.

Common garden studies have shown that amphibian populations can vary in their tolerance to climatic conditions (e.g., temperature, hydroperiod) and agrochemicals (Cothran et al. 2013; Hua et al. 2015; Johansson et al. 2001). However, these studies are difficult or impossible to scale for many species and, when implemented, may only be able test a small number of populations. To understand the extent of adaptive variation, landscape genomic surveys provide a method to identify spatial patterns in potentially adaptive genotypes, track the frequencies of allele under selection through time, and reveal the possible physiological responses behind adaptation in different environments (Forester et al. 2022).

Previous landscape genetic studies have documented links between land use and climate factors on neutral population genetic structure in amphibians (Goldberg and Waits 2010; Haugen et al. 2024; Waraniak et al. 2022). Additionally, genotype‐by‐environment analyses have found evidence of selection due to climatic gradients (Forester et al. 2025; Wu et al. 2025), but relatively few landscape genetic studies have investigated population‐wide signatures of selection due to land use and agriculture (Soria‐Ortiz and Vázquez‐Domínguez 2026). Both neutral and adaptive genetic variation carry relevance for conservation efforts, as neutral genetic variation is valuable for monitoring demographic changes and connectivity, while studies on adaptive genetic variation make assessments of evolutionary potential in response to various changes in the environment possible. When combined, information on genetic connectivity and adaptive genetic diversity can be used to assess whether populations either currently possess or can receive migrants from nearby populations with the genetic diversity necessary to keep up with changing environments, or whether interventions like genetic rescue, evolutionary rescue, or genetic provenancing may be necessary (Hoffman et al. 2021).

This study builds on previous conservation genetics work conducted on northern leopard frog ( *Rana pipiens* ), a widespread, abundant, but regionally declining amphibian (Smith and Keinath 2004; Werner 2003) in the PPR. We have previously analyzed patterns of connectivity and gene flow in *R. pipiens* in the James and Lake Oahe river basins in south‐central North Dakota (Waraniak et al. 2022). In this study, we use an expanded RAD‐seq genotyping dataset to (1) quantify the relative influence that climate, agricultural, and geographic factors have on structuring genetic diversity in *R. pipiens* populations in the PPR, and (2) identify putative genes under selection that may provide insights into the mechanisms underlying local adaptation in *R. pipiens* populations.

## 2 Methods

### 2.1 Sample Site Selection and Sample Collection

**FIGURE 1:**
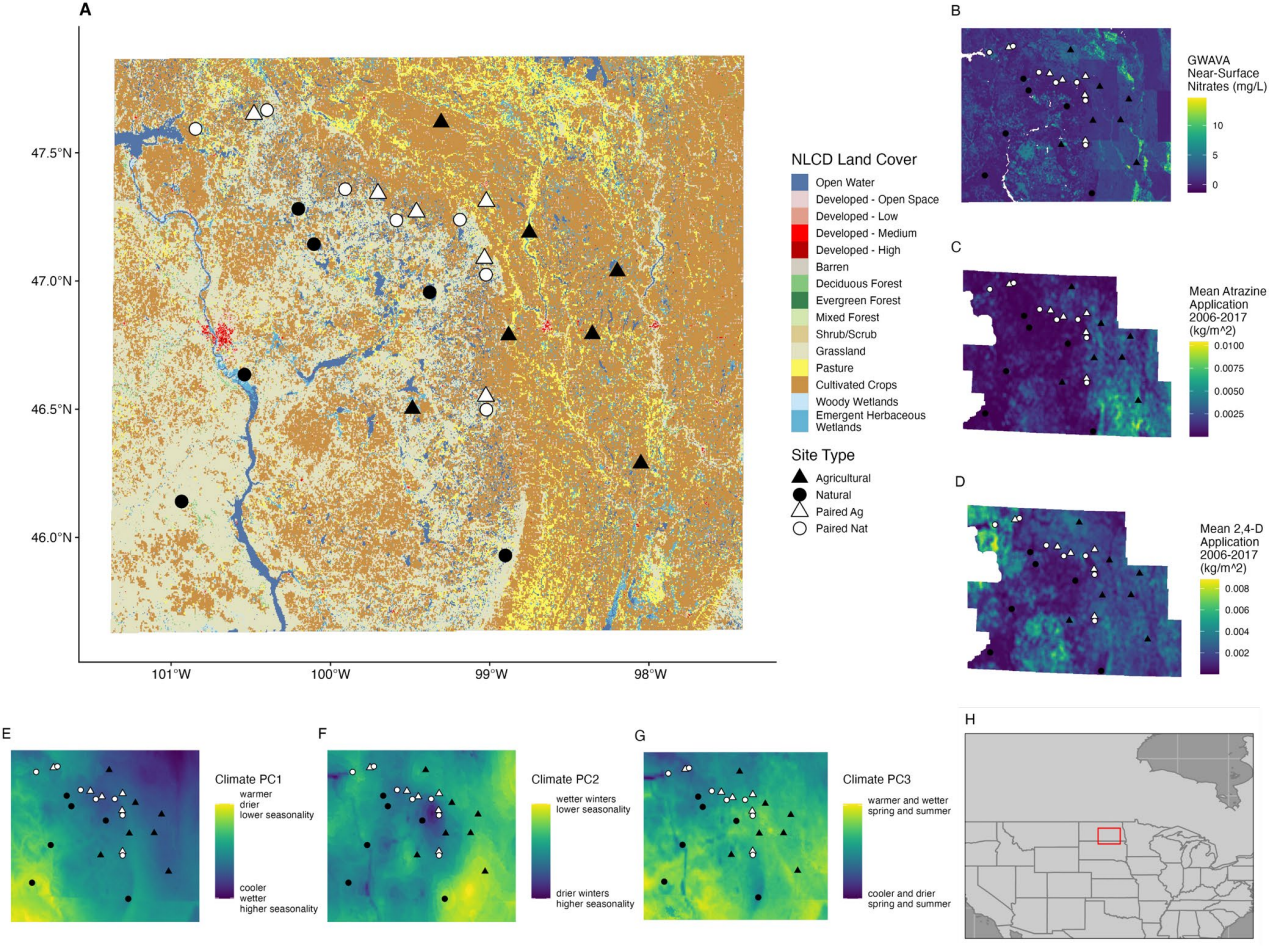
Maps of the environmental variables across the study area. From left to right in the top row, there is an inset map showing the bounds of the study area in red, the NLCD land classes, and the GWAVA‐S model of nitrate concentrations. The second row shows the estimated mean annual pesticide application from 2006 to 2017 for glyphosate, acetochlor, atrazine, and 2,4‐D. The third row shows the landscape rasters of the principal component scores of the climate variables for the first four climate principal components (Figure S1).

We conducted this study in the portions of the Lake Oahe and James River (Hydrological Unit Code HUC‐6) river basins located in North Dakota (Figure 1). Previous genetic analyses of *R. pipiens* populations in North Dakota suggested that frogs in these two river basins formed a distinct genetic cluster (Waraniak et al. 2019). We selected sample sites within the Lake Oahe and James River Basins by screening National Wetland Inventory (NWI) data for permanently ponded wetlands located on public lands (U.S. Fish and Wildlife Service 2018). We used the 2011 National Land Cover Database (NLCD; Homer et al. 2015) to calculate the proportion of land cover classified as cultivated cropland within a 5‐km buffer of each candidate wetland. Buffers of 5‐km were chosen to exceed the maximum observed distance individual *R. pipiens* have traveled in a single generation (Dole 1971). Three categories of sample sites (total *N* = 28) were chosen according to the following criteria: (i) seven sites with the least amount of cultivated cropland in the buffer area (categorized as “Natural”), (ii) seven sites with the greatest amount of cultivated cropland in the buffer area (categorized as “Agricultural”), and (iii) fourteen paired sites located between 10 and 15 km of each other with the greatest differential in the proportion of cultivated cropland in the 5‐km buffer areas (categorized as “Paired Natural” or “Paired Agricultural”). One site (or pair of sites for the paired category) that best fit the criteria for one of the categories was selected at a time. Once a site was selected, all other potential wetlands within 15 km of the selected site(s) were excluded from the candidate wetland pool. The three categories were given a random order (1–3) to start and sites were selected using a “snake draft” where the order reversed after all categories had selected a site/pair of sites (Lee and Lieu 2022).

Sample sites were visited in 2018 between late July and early August, and some with low sample sizes were resampled in 2019 during the same seasonal period. Toe‐clip tissue samples were collected from 20 individuals from each site. The sampling was conducted in late summer to target neonates that had recently metamorphosed so we could be reasonably certain that the sampled individuals originated from the target wetland. All animal handling and tissue collection was permitted by the North Dakota Game and Fish Department (License numbers GNF04736156 and GNF04910652 for 2018 and 2019, respectively) and strictly followed North Dakota State University Institutional Animal Care and Use Committee (IACUC) approved protocol #18052.

### 2.2 DNA Extraction and BestRAD Library Construction

DNA was extracted using Qiagen DNeasy Blood & Tissue Kits according to the manufacturer's protocols. Genomic libraries were constructed using the BestRAD protocol as described in Ali et al. (2016). DNA was digested using SbfI restriction enzyme prior to ligation of P1 RAD adaptors that contained one of 96 unique identifier sequences for each individual within a library. DNA from all individuals within a library was pooled and incubated with NEBNext dsDNA fragmentase for 45 min at 37°C. DNA libraries were then concentrated using Ampure XL beads and prepared for Illumina sequencing using the NEBNext Ultra II DNA Library Kit for Illumina, selecting for an average library fragment size of ~400 bp. Completed libraries were shipped to the University of Minnesota Genomics Center where they were sequenced twice on Illumina NovaSeq V1 flowcells with 150 bp paired‐end reads.

### 2.3 Genomic Data Processing

Raw genomic data was processed using Stacks v.2.66 (Catchen et al. 2013, 2017; Rochette et al. 2019). Sequence data were demultiplexed using the “process_radtags” command, assigning each sequence to the correct individual and removing sequences of poor quality. Following demultiplexing, the reads were passed through the “clone_filter” command in Stacks to remove polymerase chain reaction (PCR) duplicates introduced during the library construction process. Next, RAD loci were constructed using a reference‐based approach, aligning reads to a *R. pipiens* haploid genome (GenBank Assembly GCA_037367395.1). Initial filters with the “populations” module of Stacks screened for variant loci that appeared in at least 14 of the 28 sample sites, had coverage depths of at least 4X, and had missing genotypes in < 60% of all individuals.

Genotype data filtering was performed and threshold values for quality control filters were set using the “radiator” package in R (Gosselin 2020). Individuals with more than 50% missing data and heterozygosity values that fell outside of 0.02–0.7 were removed from the dataset. Loci that failed the following filters were removed from the dataset: (1) minor allele count below 10 (minor allele frequency < 3%), (2) average coverage less than 5X, (3) heterozygosity above 0.65, and (4) global missing data rate greater than 22%. To avoid effects of linkage, only one SNP with the highest minor allele count was kept for each locus. Loci were removed from the dataset if they fell significantly outside of Hardy–Weinberg Equilibrium (*p* < 0.05) within five or more sample sites. Basic population genetic statistics including expected and observed heterozygosity (*H*_e_ and *H*_O_, respectively) and inbreeding coefficient (*F*_IS_) were calculated for each sample site using the “hierfstat” package in R (Goudet and Jombart 2022). Two sample sites including the Long Lake National Wildlife Refuge “natural” site and the paired agriculture site near Turtle Lake were removed for having too few individuals after filtering (*N* = 2 and *N* = 3, respectively).

### 2.4 Environmental Variable Creation

Ten environmental variables were included in the genotype by environment analyses (Figure 1). The first variable was the proportion of cultivated‐crop land use based on 2011 NLCD in a 5‐km buffer around each sample site as calculated during sample site selection. Four variables estimated exposure to the four most abundant pesticides applied to crops in North Dakota: glyphosate, acetochlor, 2,4‐D, and atrazine. County‐level usage amounts (Thelin and Stone 2013; Baker and Stone 2015; Wieben 2019b) and statewide patterns of application rate by crop type (Wieben 2019a) were obtained for the years 2006 to 2017 from the National Water Quality Assessment (NAWQA) Pesticide National Synthesis Project. Spatial data of the crop types were obtained from the United States Department of Agriculture Cropland Data Layers (CDLs) for 2006 to 2017 (USDA National Agricultural Statistics Service Cropland Data Layer 2006). The CDLs were reclassified with estimates of pesticide application per cell. The ratios of pesticide applied to each crop type taken from the statewide data were held constant and applied to the county level totals of pesticide application given the amounts of each crop type in each county as calculated from the CDL for the appropriate year. A variable for nitrate exposure was calculated using the USGS Ground Water Vulnerability Assessment (GWAVA) model for near‐surface groundwater (Nolan and Hitt 2006). Values for this variable were calculated by taking the mean nitrate contamination value (mg/L) inside of a 5‐km buffer around each sample site.

Three variables were composites of climate data generated by a principal component analysis (PCA) of the 19 bioclimatic variables from the WORLDCLIM database (Fick and Hijmans 2017). The PCA was conducted on the bioclimatic variable values extracted from the locations of the 26 sample sites in the dataset and the scores of these sample sites on the first three principal component (PC) axes were kept as environmental variables for genotype by environment association analyses (GEAs). Climate PC1 explained 45% of the variation among the sample sites and was mainly associated with higher spring and summer precipitation and lower temperatures throughout the year in the eastern part of the study area compared to the southwestern portion of the study area (Figure 1 and Figure S1A). Climate PC2 explained 21% of the climate variation and was related to low winter precipitation and high precipitation seasonality along the eastern part of the Missouri Coteau (Figure 1 and Figure S1A). Climate PC3 explained 18% of the variation and was primarily related to overall warmer and wetter conditions in the southeast part of the study area compared to the northwestern portion (Figure 1 and Figure S1B).

### 2.5 Variance Partitioning

To determine the individual and combined effects of environmental and geographical variables on genetic diversity, we used redundancy analysis (RDA) models (Capblancq and Forester 2021). Redundancy analysis cannot be performed on datasets with missing data, so missing genotypes were imputed using the random forest method from the “grur” package in R (Gosselin and Archer 2019). Before the variance partitioning analysis, the 10 environmental variables were assessed for collinearity with variance inflation factors (VIF). The environmental variable with the highest VIF was removed in subsequent RDA models until all remaining constrained environmental variables had VIF < 10. The final environmental dataset included two categories: (i) climate, which included the three climate principal component variables, and (ii) agriculture, which included the percentage of nearby cultivated‐crop land‐use, estimated exposure to nitrates, 2,4‐D, and atrazine in a 5‐km buffer around each site. The full variance partitioning RDA model included these environmental variables as well as the latitude and longitude of the sample sites to quantify the amount of genetic variation explained by isolation by distance. To determine the amount of variation explained by each variable category individually, partial RDA models were run with one category (climate, agriculture, or geography) as constrained variables and the other two as conditional variables. These models were then compared to the full RDA to determine how much explained variation could be uniquely attributed to each category of environmental variable and how much explained variation was confounded between them.

### 2.6 Population Structure and Connectivity

Population structure variables were constructed using discriminant analysis of principal components (DAPC), with the clusters determined by a successive *k*‐means clustering method. Each value of *K* (from *K* = 1 to *K* = 26) was evaluated using Bayesian information criterion (BIC) and whether the assignment of individuals to *K* clusters produced at least one sample site with a majority of individuals assigned to each cluster (Puechmaille 2016; Figure S2). The *optim.a.score* function from the “adegenet” package was used to determine the number of principal component axes to retain to avoid overfitting the discriminant functions (Jombart 2008; Jombart and Ahmed 2011).

Landscape resistance and population differentiation were previously studied using an earlier version of this genomic dataset in Waraniak et al. (2022). To test whether the results of that manuscript are applicable to this updated dataset, pairwise Nei's G_ST_ and Jost's D were recalculated using the current dataset and the values were compared to the same pairs of sites reported previously using paired correlation tests. These comparisons were made using the 22 sample sites that were retained in the 2022 dataset. Additionally, the resistance distances from the model‐averaged landscape resistance models in Waraniak et al. (2022) were tested against the recalculated genetic distances using maximum likelihood population effects mixed effects models and were compared to the previous fits and null models using geographic distance as an explanatory variable. Briefly, resistance surfaces were calculated primarily using (1) 2011 NLCD land use with high resistance for open water and urban areas and moderate resistance for cultivated cropland and (2) topographic roughness with higher resistance for rougher terrain, but see Waraniak et al. (2022) for full details on what landscape variable contributed to the resistance models and how resistance models were constructed.

### 2.7 Outlier and Genotype by Environment Analysis

To identify loci potentially under selection, four outlier loci detection methods were applied to the SNP dataset. Three GEA methods related allele frequency to environmental variables including redundancy analysis (RDA) and partial redundancy analysis (pRDA) using the “vegan” package in R (Oksanen et al. 2022), *Bayenv2* (Günther and Coop 2013), and one method, *pcadapt* (Luu et al. 2017), that only identifies outliers based on background genetic structure. These approaches differ in the ways in which they account for neutral population structure and relate outliers to environmental variables. Both RDA and pRDA attempt to identify loci with allele frequency patterns that co‐vary with multivariate environmental variables, with pRDA also conditioning the genomic data on neutral population structure prior to analyzing the environmental variables. Both RDA and pRDA analyses were run because RDA may suffer from high false positive rates and pRDA from high false negative rates depending on the strength of population structure present and the degree to which neutral population structure is confounded with environmental variation. We included *Bayenv2* as a similar but statistically distinct genotype‐by‐environment association method that accounts for population structure by estimating a covariance matrix of individual genotypes as a null model and uses a Bayesian approach to find correlations between allele frequencies and individual environmental variables that are stronger than expected given the estimated population structure, which is also useful for identifying specific environmental variables that may be driving certain selective patterns. Finally, we also used the package *pcadapt* which is an outlier detection method distinct from the genotype‐by‐environment analyses in that it does not take environmental variation into account, instead identifying loci that significantly differ from population structure as quantified by PCA, which can help us identify loci which may be under selection due to environmental variables we were unable to measure.

The RDA used the same set of seven environmental variables as the variation partitioning RDA models: the three climate principal components, the nearby cultivated‐crop land‐use, and the estimated exposure to nitrates, 2,4‐D, and atrazine. Outlier loci were identified with the *rdadapt* function which calculates Malhalanobis distances from the RDA axis scores for each locus and uses a chi‐squared distribution with degrees of freedom equal to the number of RDA axes analyzed to find loci with extreme scores (Capblancq et al. 2018). Based on a screeplot of RDA axis inertia (a measure of the amount of variation explained), the scores of SNPs along the first two RDA axes were analyzed for outliers. *p*‐values obtained from the chi‐squared tests in *rdadapt* were converted to *q*‐values to provide a more robust estimation of false discovery rate (FDR), and loci with *q*‐values < 0.2 (FDR ~20%) were considered outliers putatively under selection. We used a relatively high false discovery rate as this was an exploratory analysis in a non‐model organism meant to discover potential genes for further investigation, so we were more concerned with limiting type II errors than type I errors. A partial RDA model was also run using the same genomic data and environmental variables as the RDA model described above and included additional conditioning variables of population structure. The individual cluster assignment probabilities obtained through the DAPC of population structure as described previously were used as the conditioning variables in the pRDA.

Bayenv2 was the final method used to identify loci putatively under selection and associate loci with specific environmental variables. Bayenv2 attempts to account for population structure by first computing a covariance matrix, which was calculated using a subsample of the SNP dataset that only included SNPs that did not fail Hardy–Weinberg Equilibrium tests within any populations and were pruned for long distance linkage disequilibrium. The covariance matrix was calculated using three independent runs of Bayenv2 that were then averaged together. The averaged covariance matrix is used to calculate “standardized allele frequencies” which attempt to account for population structure and uneven sample sizes. This method allows for missing data, so Bayenv2 was run on the unimputed SNP genotypes. These “standardized allele frequencies” for each locus are then regressed against the seven environmental variables retained in the RDA models, and a Bayes Factor (BF) and Spearman's rho value were calculated for each locus by environmental variable correlation test. Loci with BF > 10 and absolute value of Spearman's rho > 0.15 were considered outliers putatively under selection.

As an outlier detection method independent of environmental variables, *pcadapt* was run on the imputed genotype matrix because it cannot handle any missing data. The number of PCs to keep in the analysis to represent population structure was determined by examining the screeplot of PC eigenvalues and scatterplots of PC scores. Once the number of PCs to retain was determined (*K* = 3), *pcadapt* computed a test statistic based on Malhalanobis distances of loci along the retained principal component axes to identify loci with extreme correlations with population structure. *p*‐values from this test statistic were converted to *q*‐values and outliers with *q* < 0.2 were selected as loci putatively under selection.

### 2.8 Candidate Genes Functions

The sequences of loci that contained outlier SNPs that were detected by any of the GEA methods were then aligned to the *Rana temporaria* genome (Streicher 2021), the closest relative of *R. pipiens* with an annotated genome, with the blastn algorithm (Zhang et al. 2000) using the “discontinuous megablast” default parameters. For all loci with valid alignments, the chromosome (Sequence ID) and position on the chromosome of the alignment with the highest score was recorded and associated with the nearest protein coding gene. A gene ontology (GO) enrichment analysis was conducted by comparing the frequency of GO accession terms of the genes linked to candidate loci with the overall frequency of GO terms across all annotated genes in the *R. temporaria* genome using the *go_enrich* function in GOfuncR (Wu et al. 2021). GO enrichment analyses were run on the whole list of putatively adaptive genes identified by all GEA methods as well as separate GO analyses for lists of genes from the RDA, pRDA, and *bayenv2* tests associated with a specific environmental variable.

## 3 Results

### 3.1 Genomic Data

Raw sequence data included approximately 3.5 billion reads across 556 individuals with 6.26 ± 5.65 million reads per individual (mean ± s.d.). After STACKS and “radiator” filtering steps, the SNP dataset contained 25,587 loci and 283 individuals with an overall missing data rate of 23.6%. Sample sites at Long Lake National Wildlife Refuge and the paired agriculture site near Turtle Lake were dropped because too few individuals remained after screening by data quality filters. Sample sizes (i.e., number of *R. pipiens* individuals sampled) varied among the remaining sites after filtering (*N* = 5 to *N* = 18; Table 1).

**TABLE 1:** Information on sample sites (*N* = 27). Category refers to whether the sample site was selected for maximizing the amount of cultivated cropland in a 5‐km buffer (Agricultural), minimizing cultivated cropland (Natural), or maximizing the differential between two sites within 15 km of each other (Paired Agricultural/Natural). Sample sizes are reported for both SNP datasets used for population structure analyses and genotype by environment analyses (GEA). Observed heterozygosity (H_O_) and inbreeding coefficient (F_IS_) are reported based on the population structure dataset.

Sample site	Category	Sample size	*H*_O_	*H*_E_	*F*_is_
Albertha	Natural	14	0.075	0.092	0.187
Byersville Pag	Paired Agricultural	13	0.063	0.076	0.163
Byersville Pnat	Paired Natural	14	0.064	0.078	0.183
Chase Lake	Natural	6	0.075	0.085	0.116
Cottonwood Lakes Pag	Paired Agricultural	11	0.072	0.088	0.179
Cottonwood Lakes Pnat	Paired Natural	17	0.063	0.076	0.176
Florence Lake	Natural	7	0.048	0.058	0.175
Froelich Dam	Natural	8	0.058	0.071	0.187
Glacier Pag	Paired Agricultural	14	0.081	0.091	0.108
Glacier Pnat	Paired Natural	12	0.083	0.097	0.147
Jamestown	Agricultural	14	0.097	0.111	0.128
Jud Pag	Paired Agricultural	8	0.055	0.067	0.178
Jud Pnat	Paired Natural	16	0.097	0.111	0.131
Kensal	Agricultural	7	0.080	0.094	0.147
Logan	Agricultural	10	0.075	0.090	0.173
Lynn Pag	Paired Agricultural	13	0.078	0.093	0.158
Lynn Pnat	Paired Natural	18	0.082	0.098	0.160
MacLean Bottoms	Natural	6	0.044	0.055	0.201
Spiritwood	Agricultural	6	0.085	0.101	0.156
Sykeston	Agricultural	7	0.061	0.075	0.181
Turtle Lake Pnat	Paired Natural	7	0.048	0.06	0.156
Verona	Agricultural	12	0.073	0.089	0.176
Wallace Pag	Paired Agricultural	13	0.069	0.083	0.174
Wallace Pnat	Paired Natural	11	0.066	0.079	0.171
Wimbledon	Agricultural	11	0.072	0.086	0.170
Wing	Natural	9	0.046	0.057	0.201
Overall		283	0.070	0.083	0.165

### 3.2 Variance Partitioning Analysis

When environmental variables were examined for collinearity, variables for acetochlor and glyphosate were closely correlated with atrazine and nearby cropland, so these variables were removed. This left seven environmental variables to be used in variance partitioning and the GEAs: nearby cropland, nitrates, 2,4‐D, atrazine, and the three climate PCs.

The full RDA indicated that 3.8% of the total genetic variation could be explained by the seven environmental variables and the geographic coordinates of the sample sites (*p* = 0.002). Variance partitioning indicated that agricultural variables explained the most genetic variation; however, this was not statistically significant (37.8%, *p* = 0.33; Table 2). Climate (27.0%, *p* = 0.525) and geography representing isolation by distance (24.0%, *p* = 0.024) accounted for most of the remaining explained genetic variation. Little of the explained genetic variation was confounded among the three categories of environmental and geographic variables (11.2%, Table 2).

**TABLE 2:** Summary of variance partitioning using partial redundancy analysis models (pRDAs). Inertia analogous to variance. The full model includes all variables as constrained variables. The climate pRDA included the four bioclimatic principal component variables as constrained variables with all other variables as conditioned. The agriculture pRDA included atrazine, 2,4‐D, and nitrates as constrained variables. The geography pRDA included latitude and longitude as constrained variables. Confounded variance is the amount of inertia remaining from the full model after accounting for the inertia of each of the pRDA models.

Partial RDA models	Inertia	Pr(>F)	Proportion of explainable variance	Proportion of total variance
Full model	975	0.002*	1	0.038
Climate	263	0.525	0.270	0.010
Agriculture	369	0.330	0.378	0.014
Geography	234	0.024*	0.240	0.009
Confounded	109	—	0.112	0.004
Total unexplained	24,612	—	—	0.962
Total	25,587	—	—	1

* *p* < 0.05.

### 3.3 Population Structure

**FIGURE 2:**
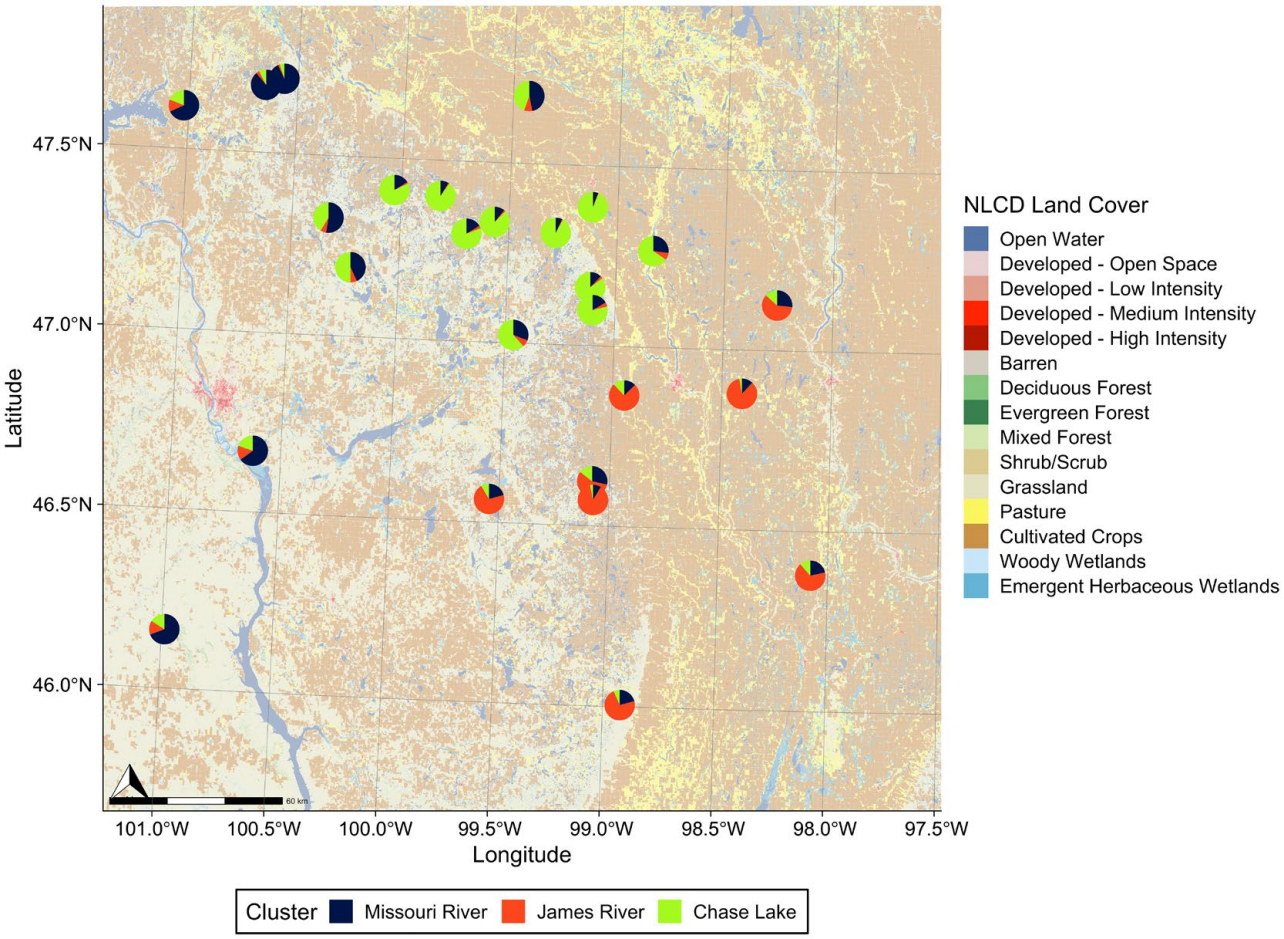
Map of the study area (top) and compoplot (bottom) of the genetic clusters delineated by the *K* = 4 DAPC clustering analysis. In the map, cumulative assignment probability to each cluster within each sample site is depicted as a pie chart.

The DAPC showed the number of genetic clusters *K* = 2 through *K* = 4 had similar BIC values. Cluster assignments of individuals for *K* = 2 and *K* = 3 produced results in which each cluster made up a majority of the assignments in at least one sample site, but this was not true for *K* = 4. For this reason and to capture the most describable population structure, *K* = 3 results were used for further analyses. DAPC results for *K* = 3 showed geographically defined clusters in the eastern portion of the study area (“James River” cluster), the eastern part of the Missouri Coteau centered around the Chase Lake National Wildlife Refuge (“Chase Lake” cluster), and in the western portion of our study area along the Missouri River (“Missouri River” cluster; Figure 2).

Pairwise genetic distance values were higher than previously calculated but strongly correlated to values from the Waraniak et al. (2022) dataset for Nei's G_ST_ (Figure S3A; *R* ^2^ = 0.131, *p* < 0.001) and Jost's D (Figure S3B; *R* ^2^ = 0.263, *p* < 0.001). Models of resistance produced by Waraniak et al. (2022) exhibited small drops in fit when comparing the recalculated genetic distances to the fits produced by the 2022 dataset (Table S1), but still outperformed null models of geographic distance, indicating that the findings in our previous analysis still explained substantial amounts of variation and were applicable to this updated dataset.

### 3.4 Outlier and Genotype by Environment Analyses

**FIGURE 3:**
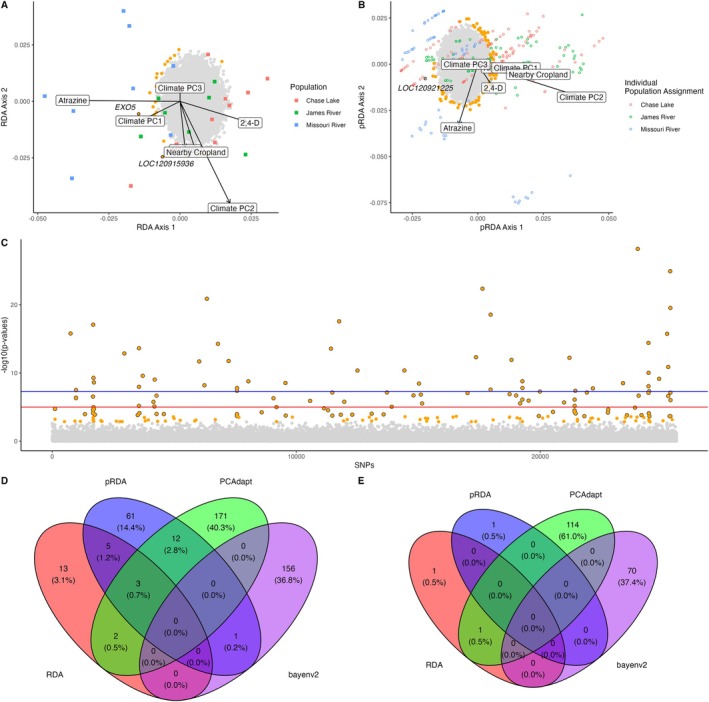
Plots of results from the outlier and genotype‐environment association (GEA) tests. RDA plots of the first two RDA axes are shown for the RDA without conditioning on population genetic structure (A) and the pRDA conditioned on genetic structure (B). Population and individual RDA scores are represented by colored boxes or small circles for the RDA (A) and pRDA (B) respectively. Environmental variables are represented by labeled arrows. A Manhattan plot is shown for the results of *pcadapt* (C). For GEA and outlier test results, candidate loci are depicted as orange points that either have a black outline with *q*‐values < 0.05 or lack a black outline with *q*‐values < 0.2. The names of select loci are labelled in italics (A–C). Venn diagrams are depicted showing overlap of outliers detected by the four genotype by environment analyses with q‐values < 0.2 and Bayes Factors > 10 (D) or q‐values < 0.05 and Bayes Factors > 20 (E). The top value is the number of outliers in each category, the bottom value is the proportion of the total number of outliers in each category.

Outlier detection tests discovered varying numbers of potential loci under selection, with RDA methods discovering fewer than *pcadapt* and *Bayenv2*. Redundancy analysis without population structure as a conditioning variable identified 23 candidate loci with *q* < 0.2 and two loci with *q* < 0.05 (Figure 3A), based on the first two RDA axes, and the partial RDA that included population structure as a conditioning variable identified 82 candidate loci with *q* < 0.2 and one locus with *q* < 0.05 (Figure 3B). Of the 23 candidate loci identified by RDA, most (18, 78.3%) were most closely associated with climate variables and five (21.7%) were associated with agricultural variables. Of the 82 candidate loci identified by pRDA, 53 (64.6%) were most closely associated with climate variables and 29 (35.4%) were associated with agricultural variables. *Bayenv2* discovered a total of 157 loci associated with environmental variables with BF > 10 and 70 loci with BF > 20. Of those loci, most (139 of 157, 88.5%; 59 of 70, 84.3%) were associated with climate variables, and fewer (21 of 157, 13.4%; 14 of 70, 20%) were associated with agricultural variables (note percentages may add up to more than 100% because some loci may be significantly associated with multiple environmental variables). *pcadapt* identified 188 candidate loci with *q*‐values < 0.2, and 115 loci with *q*‐values < 0.05 (Figure 3C).

In total, 23 loci (5.4%) out of 424 potential candidate loci were identified by more than one outlier detection test (Figure 3D,E), and 25 (6.3%) of the 399 genes associated with these loci were shared among detection methods, as several loci were mapped to the same nearest gene. Most of the overlap was shared between the RDA methods and *pcadapt*; the set of outlier loci identified by *Bayenv2* was nearly completely distinct from the other methods except for one locus shared with pRDA.

### 3.5 Candidate Loci Analysis

Valid alignments to the *R. temporaria* genome were found for 410 of the 424 candidate loci (96.7%), corresponding to 399 unique genes. GO enrichment analysis found 10 GO terms with *q* < 0.2 (Table 3). Climate PC2 was associated with the most GO terms (6, 60%), largely related to histone ubiquitination. Additionally, there were five GO terms, all related to calcium ion transport, associated with the overall list of putatively adaptive genes, driven by the inclusion of genes identified by *pcadapt*. Climate PC1, PC3, and nitrates were each associated with one GO term.

**TABLE 3:** Results of the GO enrichment analysis of over‐represented GO terms associated with candidate genes (*N* = 399) closest outlier loci. Ontology refers to the three categories of GO terms: molecular function (MF), biological process (BP), and cellular component (CC). Environmental correlates are the environmental variables that the outlier loci linked to the over‐represented GO terms are associated with based on Spearman rank correlations between allele frequencies of outlier loci and the environmental variable values. The final column shows the number of genes identified by outlier and GEA tests with the total number of genes in the reference genome annotated with the corresponding GO term.

GO term	Description	Ontology	Environmental correlate(s)	Number of genes
GO:0098703	Calcium ion import across plasma membrane	BP	—	5/25
GO:0098978	Glutamatergic synapse	CC	Climate PC1	4/24
GO:1990841	Promoter‐specific chromatin binding	MF	Climate PC2	2/6
GO:0035102	PRC1 complex	CC	Climate PC2	2/9
GO:0036353	Histone H2A‐K119 monoubiquitination	BP	Climate PC2	2/6
GO:0030424	Axon	CC	Climate PC2	5/143
GO:0005432	Calcium: sodium antiporter activity	MF	Climate PC2	2/10
GO:0043484	Regulation of RNA splicing	BP	Climate PC2	4/73
GO:0036038	MKS complex	CC	Climate PC3	2/8
GO:0005965	Protein farnesyltransferase complex	CC	Nitrates	1/2

## 4 Discussion

Patterns of putatively adaptive genetic variation in this study suggest that both climatic and land‐use factors contribute to weak, but still detectable, local adaptation in northern leopard frog populations in this part of the PPR. Populations were weakly structured within the James‐Oahe River basin, exhibiting high levels of admixture and loosely grouping into similar genetic clusters near the James River, Chase Lake, and Missouri River, as have been reported previously (Waraniak et al. 2022). Overall, land‐use variables appeared to explain more of the total genetic variation than climate, possibly reflecting how land use and topographic variables were more influential in models of connectivity for these populations than climate‐related variables in influencing the genetic structure of these populations, though the proportion of genetic variation explained by land use and climate variables was not statistically significant in RDA variance partitioning compared to genetic variation associated with geographic distance.

We found a small amount of overlap among candidate loci discovered by different GEA and outlier tests, which may be indicative that the environmental gradients we tested are not the most informative predictors of local selective pressures (Forester et al. 2018). The fact that *pcadapt*, the outlier detection test that does not compare gene frequencies to environmental variables, identified the greatest number of outliers suggests there are likely unmeasured selective gradients with greater effects on local adaptation than the ones we tested. That said, there are potentially biologically relevant patterns that can be discerned from our analysis. We found that more outlier loci putatively under selection were associated with climatic factors as opposed to agricultural land use, regardless of the GEA method used, indicating that there may be more locally adaptive genetic diversity related to climate than agriculture and land use. Additionally, we detected evidence from multiple GEA and outlier methods of putative selection on several SNPs associated with genes that play key roles in hibernation or are known to interact with pesticides. These exploratory analyses could be used to direct future studies to more explicitly test whether variation in these genes confers selective or physiological advantages under different environmental conditions.

### 4.1 Possible Climate‐Related Adaptation

Climate may impose selective effects on amphibian populations through changing temperatures (Cayuela et al. 2022; Kristin and Gvoždík 2014) or by affecting availability of breeding ponds and overwintering conditions under different seasonal precipitation regimes (Bertassello et al. 2022; Cayuela et al. 2016; O'Connor and Rittenhouse 2016; Orizaola et al. 2010). Climatic variation was associated with dozens of outlier loci linked to genes with diverse functions, but there were a few clear molecular pathways that stood out as particularly likely to be facing selective pressure.

Overwintering mortality has resulted in large die‐offs of *R. pipiens* populations in the PPR (Mushet 2010) and there could be strong selection for individuals able to survive in the low‐oxygen environments of overwintering ponds. Genes possibly related to surviving anoxic conditions during hibernation were related to climatic variables in our GEA and outlier detection tests. These genes included insulin receptor substrate 1 (IRS1) identified by RDA and pRDA tests, and genes that coded for proteins active in the glutamatergic synapse and that controlled the movement of calcium ions (Ca^2+^) were detected as outliers (Table 3 and Table S2). IRS1 is an important receptor involved in the anaerobic metabolism of other overwintering ectotherms (Szereszewski and Storey 2018). Glutamatergic synapses are calcium‐dependent glutamate receptors active in the nervous system, particularly responsible for fast synaptic transmission. Overexcitation of glutamatergic synapses and a buildup of Ca^2+^ caused by hypoxic conditions can lead to nervous system dysregulation (Bueschke et al. 2024). Calcium‐transport proteins could help remove excess Ca^2+^ from cells and prevent neuron dysregulation during hypoxia, and changes in the glutamatergic synapse itself may reduce sensitivity to Ca^2+^ and lower the likelihood that synapses would become overexcited (Bueschke et al. 2024).

Chromatin remodeling also appeared to be one of the most prominent molecular pathways facing selection due to climatic variation. Chromatin remodeling is a common epigenetic response to a wide variety of environmental stressors, including chronic temperature stress and anoxia (Ingelson‐Filpula et al. 2022). Additionally, in amphibians, histone modifications are important mechanisms inducing transcriptional changes during overwintering (Hawkins and Storey 2018; Ingelson‐Filpula et al. 2022) and metamorphosis (Grimaldi et al. 2013; Kuecks‐Winger et al. 2025). Ubiquitination of histone H2A appears to be the main chromatin modification under selection in this system, with genes related to the polycomb group complex (*pcgf2*) which catalyzes the ubiquitination of histones, multiple ubiquitin ligases (LOC120924746, LOC120930265, LOC120930496, LOC120932431, *ubr3*) identified by single outlier tests, and histone H2A itself (LOC120932405) identified by both *pcadapt* and RDA.

### 4.2 Outlier Genes Associated With Agrochemicals

Comparatively fewer putatively adaptive loci were associated with agricultural land‐use variables versus climatic variables. However, some loci potentially linked to genes active in xenobiotic pathways were identified. Two different cytochrome P450 enzymes (LOC120926886, LOC120941049) and a related cytochrome b5 enzyme (LOC12091684) were associated with atrazine and 2,4‐D exposure. Cytochrome P450 enzymes are primarily responsible for metabolizing biotoxins and are consistently found as some of the most upregulated genes in transcriptomic experiments involving exposures of animals to atrazine and other chemical pesticides (Xia et al. 2016; Fu et al. 2013; Abass et al. 2012; Suzawa and Ingraham 2008; Khan et al. 1998). Another gene that interacts with cytochrome P450, nuclear receptor SF‐1 (*nr5a1*) was also highlighted by both pRDA and *pcadapt*, though it was associated most closely with Climate PC2 in the pRDA. Nuclear receptor SF‐1 interacts directly with atrazine, which may facilitate atrazine‐induced endocrine disruption in reproductive organs, leading to toxicity and developmental abnormalities in female amphibians and other animals (Zhao et al. 2024; Suzawa and Ingraham 2008; Fan et al. 2007).

### 4.3 Potential Analytical Biases and Data Limitations

Due to the exploratory nature of the analyses presented in this manuscript, caution should be taken when interpreting claims of evidence for selection on specific genes. While the overall patterns of selection in our dataset appear relatively robust, namely that there appears to be greater adaptive genetic diversity present related to climatic factors when compared to agricultural factors, the exploratory nature of these analyses and relatively relaxed criteria we used to identify candidate loci mean that a high proportion of the identified candidate loci could be false positives. Most of the genes and pathways highlighted in the discussion are supported by multiple methods or are represented by numerous genes identified by different outlier detection methods and have a higher degree of confidence than other parts of our dataset; however, the interpretation of selection applied to any single gene in our results, especially those identified only by one outlier detection method, should be approached with caution.

Additionally, these results are correlative and additional evidence is required to establish causal links between specific environmental variables and putatively adaptive loci. The methods used in this study give contrasting environmental associations for some loci, which could be spurious due to spatial correlations in environmental variables or interactive selective pressures induced by multiple environmental variables. It cannot be determined from our analysis whether the adaptive variation associated with different climate and agricultural land‐use variables is mutually adaptive or if the selection associated with some variables may reduce fitness and adaptive capacity to changes in other variables (Carrasco et al. 2021). Climatic factors can have interactive effects with toxicity caused by agrochemicals (Carneiro et al. 2023; Baier et al. 2016), potentially causing intense or unpredictable changes to selective regimes. Future research could focus on how selection imposed by changing climate regimes and agrochemical exposure interact, and how key biomolecular systems respond to multiple stressors.

These results may also be insensitive for detection of relatively uncommon alleles that may be under selection. Uneven sample sizes in combination with imputation of genotypes could mean that some rare genotypes were under‐represented in our dataset (Rutkowski et al. 2013). Sample sites with fewer individuals are more likely to lack information on lower frequency alleles, and because imputation was informed by the allele frequencies already detected in the sample site of origin, the imputation method would be biased toward the alleles already observed in a sample site. This would have downstream implications where relatively rare alleles that were actually present in a site were not identified, possibly leading to a higher number of spurious results in GEAs: both false negatives for rare alleles that may only appear in areas where the rare allele is being positively selected for but were not properly represented by imputation, and false positives for alleles that are in fact relatively evenly distributed throughout the landscape but were missed in sample sites with lower sample sizes and higher missing genotypes.

## 5 Conclusion

While climate and land‐use environmental factors had relatively small effects on population structure and overall genomic variation in this *R. pipiens* population in the PPR, outlier detection tests found putatively adaptive genomic variation, most notably associated with overwintering physiology and xenobiotic metabolism. High levels of gene flow and large population sizes characteristic of *R. pipiens* in this part of the PPR, coupled with the presence of adaptive diversity, imply that these *R. pipiens* theoretically have a high degree of adaptive capacity (Waraniak et al. 2022). However, questions remain on (1) whether the adaptive diversity present is strong enough for evolutionary rescue (*sensu* Bell and Gonzalez 2009) to be viable for these populations, (2) if selection from multiple stressors is acting synergistically or antagonistically, and (3) if landscape resistance from agricultural land use, as has been previously reported (Waraniak et al. 2022), may impede the spread of adaptive alleles through the breeding populations where they be most beneficial. Our study indicates further research investigating differences in the highlighted biomolecular and physiological responses of putatively locally adapted populations to multiple stressors and monitoring of genetic variation through changing environmental conditions would inform the role of adaptation on the persistence of amphibian populations in agriculturally dominated landscapes.

## Funding

Funding was provided by the US Geological Survey Climate and Land Use Change Research and Development Program (Grant #G16AC00271 and #G19AC00210), the North Dakota Water Resources Research Institute, and North Dakota Game and Fish.

## Conflicts of Interest

The authors declare no conflicts of interest.

## Supporting information


**Figure S1:** Principal component of analysis of the 19 Worldclim bioclimatic variables at the 26 sample sites. Sample sites are represented by black dots, loadings of the bioclimatic variables along each axis are represented by red arrows and text.
**Figure S2:** Metrics used to select the number of *K* clusters for the discriminant analysis of principal components on neutral population structure. BIC values for *K* = 2 and *K* = 4 are similar for the *k*‐means analysis.
**Figure S3:** Comparison of genetic distances calculated from the dataset presented in Waraniak et al. (2022) to the genetic distances calculated from the current version of this dataset. Two genetic distances used to assess resistance surfaces were calculated, Nei's G_ST_ (A) and Jost's D (B).
**Table S1:** Results of maximum likelihood population effects random effects models comparing the fits of resistance models using recalculated genetic distances to the genetic distances from Waraniak et al. (2022) and null models of geographic distance. Model fits were assessed using ∆BIC and marginal *R* ^2^ for two genetic distances, Nei's G_ST_ and Jost's D.
**Table S2:** List of genes with gene symbol and gene name from the *Rana temporaria* reference genome that were aligned to loci identified as putatively under selection in *Rana pipiens* from our study area. Genes associate with SNPs identified by multiple genotype by association and outlier tests are listed first.

## Data Availability

The data that support the findings of this study are openly available in Dryad at https://doi.org/10.5061/dryad.bk3j9kdr1
