# Supplementary material for: Patterns of Local Adaptation in the Northern Leopard Frog (Rana pipiens) From a Region Undergoing Rapid Climate and Land‐Use Change
Source: Evol Appl. 2026 Jul 9;19(7):e70298. doi: 10.1111/eva.70298 (PMC13351322; doi:10.1111/eva.70298)
Supplement: Supplementary file 1 — Figure S1: Principal component of analysis of the 19 Worldclim bioclimatic variables at the 26 sample sites. Sample sites are represented by black dots, loadings of the bioclimatic variables along each axis are represented by red arrows and text. Figure S2: Metrics used to select the number of K clusters for the discriminant analysis of principal components on neutral population structure. BIC values for K = 2 and K = 4 are similar for the k‐means analysis. Figure S3: Comparison of genetic distances calculated from the dataset presented in Waraniak et al. (2022) to the genetic distances calculated from the current version of this dataset. Two genetic distances used to assess resistance surfaces were calculated, Nei's GST (A) and Jost's D (B). Table S1: Results of maximum likelihood population effects random effects models comparing the fits of resistance models using recalculated genetic distances to the genetic distances from Waraniak et al. (2022) and null models of geographic distance. Model fits were assessed using ∆BIC and marginal R 2 for two genetic distances, Nei's GST and Jost's D. Table S2: List of genes with gene symbol and gene name from the Rana temporaria reference genome that were aligned to loci identified as putatively under selection in Rana pipiens from our study area. Genes associate with SNPs identified by multiple genotype by association and outlier tests are listed first. [file EVA-19-e70298-s001.zip › Suppl_Tables_Figures.docx]

Fig S1. Principal component of analysis of the 19 Worldclim bioclimatic variables at the 26 sample sites. Sample sites are represented by black dots, loadings of the bioclimatic variables along each axis are represented by red arrows and text.

Fig S2. Metrics used to select the number of K clusters for the discriminant analysis of principal components on neutral population structure. BIC values for K=2 and K=4 are similar for the k-means analysis.

Fig S3. Comparison of genetic distances calculated from the dataset presented in Waraniak et al. (2022) to the genetic distances calculated from the current version of this dataset. Two genetic distances used to assess resistance surfaces were calculated, Nei’s G_ST_ (A) and Jost’s D (B).

Table S1. Results of maximum likelihood population effects random effects models comparing the fits of resistance models using recalculated genetic distances to the genetic distances from Waraniak et al. (2022) and null models of geographic distance. Model fits were assessed using ∆BIC and marginal R^2^ for two genetic distances, Nei’s G_ST_ and Jost’s D.

| Model | ∆BIC | Marginal R^2^ |
| --- | --- | --- |
| G_ST_ Models |  |  |
| Original (2022) resistance model fit | 0 | 0.178 |
| Recalculated resistance model fit | 22.5 | 0.116 |
| Recalculated geographic model fit | 84.1 | 0.058 |
| D_J_ Models |  |  |
| Original (2022) resistance model fit | 0 | 0.441 |
| Recalculated resistance model fit | 43.0 | 0.484 |
| Recalculated geographic model fit | 93.0 | 0.223 |

Table S2. List of genes with gene symbol and gene name from the *Rana temporaria* reference genome that were aligned to loci identified as putatively under selection in *Rana pipiens* from our study area. Genes associate with SNPs identified by multiple genotype by association and outlier tests are listed first.

| Gene Symbol | Gene Name | GEA Associations |
| --- | --- | --- |
| AARS2 | alanine--tRNA ligase, mitochondrial | pRDA (Climate PC1), pcadapt |
| CDH18 | cadherin-18 | pcadapt, pRDA (Climate PC2) |
| CUL3 | cullin-3 | RDA (Climate PC3), pRDA (Climate PC1) |
| DAPP1 | dual adapter for phosphotyrosine and 3-phosphotyrosine and 3-phosphoinositide | RDA (Climate PC3),  pRDA (Cropland) |
| EXO5 | exonuclease V | pRDA (Cropland),  RDA (Atrazine) |
| IRS1 | insulin receptor substrate 1 | RDA (Climate PC3), pRDA (Climate PC1) |
| KCNQ4 | potassium voltage-gated channel subfamily KQT member 4 | RDA (Climate PC3),  pRDA (Cropland) |
| LOC120913207 | uncharacterized LOC120913207 | pcadapt, pRDA (Climate PC1), RDA (Nitrates) |
| LOC120915936 | trypsin-3-like | pcadapt, RDA (Climate PC1), pRDA (Climate PC1) |
| LOC120920963 | protein farnesyltransferase subunit beta-like | pcadapt, pRDA (Nitrates) |
| LOC120924717 | early activation antigen CD69-like | pcadapt, pRDA (2,4-D) |
| LOC120924993 | vomeronasal type-2 receptor 26-like | pcadapt, pRDA (Climate PC1) |
| LOC120925112 | vomeronasal type-2 receptor 26-like | pcadapt, pRDA (Cropland) |
| LOC120927803 | uncharacterized protein LOC120927803 | pcadapt, pRDA (Atrazine), RDA (Climate PC1) |
| LOC120928546 | uncharacterized LOC120928546 | pcadapt, pRDA (Climate PC3) |
| LOC120932405 | core histone macro-H2A.1 | pcadapt, RDA (2,4-D) |
| LOC120933832 | NACHT, LRR and PYD domains-containing protein 3-like | pcadapt, pRDA (Atrazine) |
| LOC120933840 | uncharacterized gene | pcadapt, pRDA (2,4-D) |
| LOC120940763 | uncharacterized LOC120940763 | bayenv2 (Climate PC1), pcadapt, pRDA (Nitrates) |
| LOC120941201 | uncharacterized protein LOC120941201 | pcadapt, pRDA (2,4-D) |
| LRP8 | low-density lipoprotein receptor-related protein 8 | pRDA (Climate PC3), RDA (Climate PC3) |
| MAN2A1 | alpha-mannosidase 2 | pcadapt, RDA (Climate PC3) |
| MTFR1L | mitochondrial fission regulator 1-like | pcadapt, pRDA (Climate PC3) |
| NR5A1 | steroidogenic factor 1 | pcadapt, pRDA (Climate PC2) |
| THEG | testicular haploid expressed gene protein | bayenv2 (Climate PC3), pRDA (2,4-D) |
| ABCA12 | ATP-binding cassette sub-family A member 12 | bayenv2 (Climate PC3) |
| ABCB9 | ATP-binding cassette sub-family B member 9 | pRDA (Climate PC2) |
| ABHD3 | phospholipase ABHD3 | bayenv2 (2,4-D) |
| ABLIM1 | actin-binding LIM protein 1 | Pcadapt |
| ABLIM3 | actin-binding LIM protein 3 | bayenv2 (Climate PC1) |
| ACIN1 | apoptotic chromatin condensation inducer in the nucleus | bayenv2 (Climate PC2) |
| ADCY1 | adenylate cyclase type 1 | bayenv2 (Climate PC3) |
| ADGRL4 | adhesion G protein-coupled receptor L4 | bayenv2 (Climate PC3) |
| ADGRV1 | adhesion G protein-coupled receptor V1 | bayenv2 (Climate PC3) |
| ADPRS | ADP-ribose glycohydrolase ARH3 | pcadapt |
| AFAP1L1 | actin filament-associated protein 1-like 1 | pcadapt |
| AGBL4 | cytosolic carboxypeptidase 6 | bayenv2 (Climate PC1, PC3) |
| AHCYL1 | S-adenosylhomocysteine hydrolase-like protein 1 | pRDA (Climate PC1) |
| AHCYL2 | adenosylhomocysteinase 3 | bayenv2 (Climate PC2) |
| AKAP13 | A-kinase anchor protein 13 | pcadapt |
| ALCAM | CD166 antigen | bayenv2 (Climate PC2) |
| ANKRD50 | ankyrin repeat domain-containing protein 50 | bayenv2 (Climate PC1) |
| ANO2 | anoctamin-2 | pRDA (Climate PC1) |
| ANTXR2 | anthrax toxin receptor 2 | bayenv2 (Climate PC3) |
| ANXA3 | annexin A3 | bayenv2 (Atrazine) |
| APAF1 | apoptotic protease-activating factor 1 | bayenv2 (Climate PC3) |
| ARPP19 | cAMP-regulated phosphoprotein 19 | bayenv2 (Climate PC3) |
| ARRDC4 | arrestin domain-containing protein 4 | bayenv2 (Climate PC3) |
| ASS1 | argininosuccinate synthase | RDA (Climate PC1) |
| ASXL2 | putative Polycomb group protein ASXL2 | bayenv2 (Climate PC2) |
| B3GALT1 | beta-1,3-galactosyltransferase 1 | pRDA (Climate PC1) |
| B9D2 | B9 domain-containing protein 2 | bayenv2 (Climate PC3) |
| BAG1 | BAG family molecular chaperone regulator 1 | pcadapt |
| BEND7 | BEN domain-containing protein 7 | pcadapt |
| BTBD11 | ankyrin repeat and BTB/POZ domain-containing protein BTBD11 | pRDA (Climate PC1) |
| BUD23 | probable 18S rRNA (guanine-N(7))-methyltransferase | pRDA (Climate PC1) |
| C3H12orf73 | protein BRAWNIN | bayenv2 (Climate PC2) |
| C5H7orf25 | UPF0415 protein C7orf25 homolog | pcadapt |
| C8H10orf71 | cardiac-enriched FHL2-interacting protein | bayenv2 (Climate PC3) |
| CACNA1C | voltage-dependent L-type calcium channel subunit alpha-1C | pRDA (Climate PC2) |
| CACNA1D | voltage-dependent L-type calcium channel subunit alpha-1D | pcadapt |
| CADPS | calcium-dependent secretion activator 1 | pRDA (Climate PC1) |
| CALCB | calcitonin gene-related peptide 2 | bayenv2 (2,4-D) |
| CAPN15 | calpain-15 | pRDA (Climate PC3) |
| CC2D2A | coiled-coil and C2 domain-containing protein 2A | pcadapt |
| CCDC62 | coiled-coil domain-containing protein 62 | pcadapt |
| CCNB3 | G2/mitotic-specific cyclin-B3 | pcadapt |
| CCNK | cyclin-K | pcadapt |
| CCSER2 | serine-rich coiled-coil domain-containing protein 2 | pcadapt |
| CDK17 | cyclin-dependent kinase 17 | pcadapt |
| CEBPD | CCAAT/enhancer-binding protein delta | bayenv2 (Climate PC3) |
| CEP162 | centrosomal protein of 162 kDa | bayenv2 (Climate PC3) |
| CERS1 | ceramide synthase 1 | pRDA (Climate PC1) |
| CHD9 | chromodomain-helicase-DNA-binding protein 9 | pcadapt |
| CHRM2 | muscarinic acetylcholine receptor M2 | pcadapt |
| COASY | bifunctional coenzyme A synthase | pcadapt |
| CORIN | atrial natriuretic peptide-converting enzyme | pcadapt |
| CPM | carboxypeptidase M | pcadapt |
| CRB1 | protein crumbs homolog 1 | pRDA (Climate PC2) |
| DACH1 | dachshund homolog 1 | bayenv2 (Climate PC2) |
| DCTN1 | dynactin subunit 1 | bayenv2 (Climate PC2) |
| DKK1 | dickkopf-related protein 1 | Pcadapt |
| DLG5 | disks large homolog 5 | Pcadapt |
| DLGAP3 | disks large-associated protein 3 | bayenv2 (Climate PC3) |
| DLX2 | homeobox protein DLX-2 | pcadapt |
| DNHD1 | dynein heavy chain domain-containing protein 1 | pcadapt |
| DNM1L | dynamin-1-like protein isoform | pcadapt |
| DPP3 | dipeptidyl peptidase 3 | pcadapt |
| EDC3 | enhancer of mRNA-decapping protein 3 | bayenv2 (Climate PC3) |
| EEPD1 | endonuclease/exonuclease/phosphatase family domain-containing protein 1 | bayenv2 (2,4-D, Nitrates) |
| EIF1AX | eukaryotic translation initiation factor 1A, X-chromosomal | pcadapt |
| ELAVL1 | ELAV-like protein 1 | pcadapt |
| ENPP7 | ectonucleotide pyrophosphatase/ phosphodiesterase family member 7 | bayenv2 (Climate PC3) |
| EP300 | histone acetyltransferase p300 | bayenv2 (Climate PC3) |
| EVI5L | EVI5-like protein | pRDA (Climate PC1) |
| FAM222A | protein FAM222A | pcadapt |
| FAM43A | protein FAM43A | pcadapt |
| FAM83B | protein FAM83B | bayenv2 (Climate PC2) |
| FAM89A | protein FAM89A | pcadapt |
| FASLG | tumor necrosis factor ligand superfamily member 6 | pcadapt |
| FAT1 | protocadherin Fat 1 | bayenv2 (Climate PC3) |
| FGD3 | FYVE, RhoGEF and PH domain-containing protein 3 | RDA (Climate PC3) |
| FGFR2 | fibroblast growth factor receptor 2 | pcadapt |
| FPGT | fucose-1-phosphate guanylyltransferase | pRDA (Climate PC1) |
| FRK | tyrosine-protein kinase FRK | pcadapt |
| FRRS1 | ferric-chelate reductase 1 | bayenv2 (Climate PC2) |
| FUT9 | 4-galactosyl-N-acetylglucosaminide 3-alpha-L-fucosyltransferase 9 | pRDA (Climate PC3) |
| FXR1 | fragile X mental retardation syndrome-related protein 1 | bayenv2 (Climate PC2) |
| GABRB2 | gamma-aminobutyric acid receptor subunit beta-1 | pcadapt |
| GADL1 | acidic amino acid decarboxylase GADL1 | pcadapt |
| GALM | galactose mutarotase | pcadapt |
| GALNT1 | polypeptide N-acetylgalactosaminyltransferase 1 | pcadapt |
| GAS2L1 | GAS2-like protein 1 | pcadapt |
| GC | vitamin D-binding protein | pcadapt |
| GDPD5 | glycerophosphodiester phosphodiesterase domain-containing protein 5 | pcadapt |
| GFRA1 | GDNF family receptor alpha-1 | pRDA (Climate PC1) |
| GLRA1 | glycine receptor subunit alpha-1 | bayenv2 (Climate PC3) |
| GLRX2 | glutaredoxin 2 | bayenv2 (Cropland) |
| GMDS | GDP-mannose 4,6 dehydratase | bayenv2 (Climate PC2) |
| GNG4 | guanine nucleotide-binding protein G(I)/G(S)/G(O) subunit gamma-4 | pcadapt |
| GRIN2A | glutamate receptor ionotropic, NMDA 2A | bayenv2 (Cropland, Atrazine) |
| GRIPAP1 | GRIP1-associated protein 1 | pRDA (Climate PC1) |
| GUCY2D | retinal guanylyl cyclase 1 | bayenv2 (Climate PC3) |
| HEATR5B | HEAT repeat-containing protein 5B | RDA (Climate PC3) |
| HIF1AN | hypoxia-inducible factor 1-alpha inhibitor | pcadapt |
| HS1BP3 | HCLS1-binding protein 3 | pcadapt |
| HSD17B4 | peroxisomal multifunctional enzyme type 2 | pcadapt |
| IFNGR1 | interferon gamma receptor 1 | pRDA (Climate PC1) |
| IKZF2 | zinc finger protein Helios | pcadapt |
| IMPA1 | inositol monophosphatase 1 | pRDA (2,4-D) |
| IP6K2 | inositol hexakisphosphate kinase 2 | bayenv2 (Climate PC1) |
| IPO11 | importin-11 | bayenv2 (2,4-D) |
| IQSEC1 | IQ motif and SEC7 domain-containing protein 1 | pcadapt |
| IRX4 | iroquois-class homeodomain protein IRX-4 | RDA (Climate PC3) |
| ITGB6 | integrin beta-6 | pcadapt |
| JAK3 | tyrosine-protein kinase JAK3 | bayenv2 (Climate PC2) |
| KAT6B | histone acetyltransferase KAT6B | pRDA (Cropland) |
| KCNF1 | potassium voltage-gated channel subfamily F member 1 | bayenv2 (Climate PC1) |
| KCNMB1 | calcium-activated potassium channel subunit beta-1 | pcadapt |
| KCNS2 | potassium voltage-gated channel subfamily S member 2 | bayenv2 (Climate PC3) |
| KDM4B | lysine-specific demethylase 4B | pcadapt |
| KHDRBS2 | KH domain-containing, RNA-binding, signal transduction-associated protein 2 | bayenv2 (Climate PC2) |
| KLF15 | Krueppel-like factor 15 | bayenv2 (Climate PC1) |
| KTN1 | kinectin | bayenv2 (Climate PC2) |
| LARGE1 | LARGE xylosyl- and glucuronyltransferase 1 | bayenv2 (Climate PC3) |
| LEF1 | lymphoid enhancer-binding factor 1 | pcadapt |
| LMBRD2 | G-protein coupled receptor-associated protein LMBRD2 | pcadapt |
| LMTK3 | serine/threonine-protein kinase LMTK3 | bayenv2 (Climate PC2) |
| LOC120908951 | prosaposin | pRDA (Climate PC1) |
| LOC120909063 | nuclear receptor 2C2-associated protein | pRDA (2,4-D) |
| LOC120909077 | uncharacterized protein LOC120909077 | bayenv2 (Climate PC3) |
| LOC120909156 | glutathione S-transferase P 1-like | pcadapt |
| LOC120909175 | oxysterol-binding protein 1-like | bayenv2 (Climate PC3) |
| LOC120909285 | membrane-spanning 4-domains subfamily A member 4D-like | RDA (Climate PC3) |
| LOC120909314 | membrane-spanning 4-domains subfamily A member 4A-like | pcadapt |
| LOC120909605 | zinc finger protein 585A-like | pcadapt |
| LOC120910374 | alpha-1-macroglobulin-like | pcadapt |
| LOC120910703 | uncharacterized LOC120910703 | pcadapt |
| LOC120913437 | mRNA decay activator protein ZFP36-like | pcadapt |
| LOC120913440 | phospholipid-transporting ATPase IC-like | pcadapt |
| LOC120913800 | disabled homolog 2-interacting protein | pcadapt |
| LOC120914120 | uncharacterized LOC120914120 | pcadapt |
| LOC120914250 | neural cell adhesion molecule L1-like | bayenv2 (Climate PC2) |
| LOC120914831 | U6atac minor spliceosomal RNA | bayenv2 (Climate PC3) |
| LOC120916240 | Friend leukemia integration 1 transcription factor-like | pRDA (Climate PC3) |
| LOC120916466 | carcinoembryonic antigen-related cell adhesion molecule 10-like | bayenv2 (Climate PC1) |
| LOC120916588 | GTPase HRas-like | bayenv2 (Climate PC3) |
| LOC120916804 | cytochrome b5-like | bayenv2 (Climate PC3, Atrazine) |
| LOC120916874 | uncharacterized gene | pcadapt |
| LOC120918475 | bifunctional heparan sulfate N-deacetylase/N-sulfotransferase 3-like | bayenv2 (Atrazine) |
| LOC120918584 | uncharacterized LOC120918584 | bayenv2 (Climate PC2) |
| LOC120918606 | protein-glutamine gamma-glutamyltransferase E-like | pcadapt |
| LOC120918773 | bladder cancer-associated protein | bayenv2 (Climate PC3) |
| LOC120919374 | uncharacterized LOC120919374 | bayenv2 (Climate PC2) |
| LOC120919385 | uncharacterized protein LOC120919385 | bayenv2 (Climate PC1) |
| LOC120919436 | uncharacterized LOC120919436 | bayenv2 (Climate PC3) |
| LOC120919760 | 5.8S ribosomal RNA | pcadapt |
| LOC120920459 | toll-like receptor 2 | pcadapt |
| LOC120920856 | uncharacterized protein LOC120920856 | bayenv2 (Climate PC3) |
| LOC120921225 | 40S ribosomal protein S6-like | pRDA (Climate PC3) |
| LOC120921561 | uncharacterized LOC120921561 | bayenv2 (Climate PC2) |
| LOC120921619 | uncharacterized protein LOC120921619 | pcadapt |
| LOC120921701 | LRP2-binding protein | RDA (Climate PC1) |
| LOC120921761 | uncharacterized LOC120921761 | pcadapt |
| LOC120921763 | uncharacterized LOC120921763 | pcadapt |
| LOC120924431 | uncharacterized protein LOC120924431 | pcadapt |
| LOC120924690 | C-type lectin domain family 10 member A-like | pcadapt |
| LOC120924740 | uncharacterized LOC120924740 | pcadapt |
| LOC120924746 | E3 ubiquitin-protein ligase TRIM8-like | pcadapt |
| LOC120924803 | uncharacterized LOC120924803 | pcadapt |
| LOC120924807 | proteinase-activated receptor 1-like | pcadapt |
| LOC120924855 | uncharacterized LOC120924855 | pcadapt |
| LOC120924934 | small nuclear ribonucleoprotein F-like | pcadapt |
| LOC120924987 | uncharacterized LOC120924987 | pRDA (Climate PC1) |
| LOC120924991 | uncharacterized LOC120924991 | pcadapt |
| LOC120925116 | vomeronasal type-2 receptor 26-like | pcadapt |
| LOC120925155 | vomeronasal type-2 receptor 26-like | pcadapt |
| LOC120925250 | uncharacterized gene | pcadapt |
| LOC120925541 | vomeronasal type-2 receptor 26-like | pcadapt |
| LOC120925710 | U1 spliceosomal RNA | pRDA (2,4-D) |
| LOC120925747 | U2 spliceosomal RNA | bayenv2 (Atrazine) |
| LOC120925854 | uncharacterized gene | pcadapt |
| LOC120926886 | cytochrome P450 4F22 | bayenv2 (2,4-D) |
| LOC120927074 | uncharacterized protein C6orf132-like | pcadapt |
| LOC120927407 | vomeronasal type-2 receptor 26-like | pcadapt |
| LOC120927408 | uncharacterized gene | bayenv2 (Climate PC2) |
| LOC120927948 | E3 SUMO-protein ligase ZBED1-like | pRDA (Climate PC1) |
| LOC120928131 | vomeronasal type-2 receptor 26-like | pcadapt |
| LOC120928170 | uncharacterized LOC120928170 | pcadapt |
| LOC120928269 | zinc finger MYM-type protein 1-like | bayenv2 (Climate PC2) |
| LOC120928353 | golgin subfamily A member 6-like protein 22 | pcadapt |
| LOC120928396 | uncharacterized protein LOC120928396 | pcadapt |
| LOC120928457 | uncharacterized gene | pRDA (Climate PC2) |
| LOC120928543 | uncharacterized LOC120928543 | pRDA (Atrazine) |
| LOC120928552 | protein ALP1-like | pcadapt |
| LOC120928556 | uncharacterized LOC120928556 | pcadapt |
| LOC120928653 | uncharacterized LOC120928653 | pRDA (Climate PC1) |
| LOC120928758 | ecto-ADP-ribosyltransferase 5-like | pcadapt |
| LOC120928810 | cholecystokinin receptor-like | bayenv2 (Climate PC3) |
| LOC120928899 | teneurin-4-like | bayenv2 (Climate PC2) |
| LOC120929036 | vomeronasal type-2 receptor 26-like | pRDA (Nitrates) |
| LOC120929057 | solute carrier family 35 member F2-like | pcadapt |
| LOC120929110 | serine/threonine-protein kinase Nek5-like | bayenv2 (Climate PC3) |
| LOC120929666 | uncharacterized LOC120929666 | bayenv2 (Climate PC2) |
| LOC120929924 | uncharacterized gene | pcadapt |
| LOC120930265 | E3 ubiquitin-protein ligase Jade-2-like | pcadapt |
| LOC120930376 | nucleoside diphosphate kinase A2 | pcadapt |
| LOC120930393 | uncharacterized protein LOC120930393 | pcadapt |
| LOC120930395 | nodal homolog 2-A-like | bayenv2 (Climate PC1) |
| LOC120930448 | secreted RxLR effector protein 161-like | pcadapt |
| LOC120930453 | uncharacterized protein LOC120930453 | bayenv2 (Climate PC3) |
| LOC120930496 | E3 ubiquitin/ISG15 ligase TRIM25-like | bayenv2 (Climate PC3) |
| LOC120930541 | nodal homolog 2-B-like | bayenv2 (Climate PC3) |
| LOC120931248 | ras and EF-hand domain-containing protein-like | pcadapt |
| LOC120931739 | guanine nucleotide-binding protein G(i) subunit alpha-1 | pRDA (Cropland) |
| LOC120932127 | tyrosine 3-monooxygenase-like | pcadapt |
| LOC120932431 | ubiquitin-conjugating enzyme E2 B | bayenv2 (Climate PC3) |
| LOC120932441 | deoxycytidine kinase 2-like | pcadapt |
| LOC120932694 | sodium- and chloride-dependent GABA transporter 2-like | pRDA (Climate PC1) |
| LOC120932869 | solute carrier family 23 member 1-like | pcadapt |
| LOC120932877 | WAS/WASL-interacting protein family member 1-like | bayenv2 (Climate PC3) |
| LOC120933190 | G protein-coupled receptor kinase 5-like | bayenv2 (Climate PC1) |
| LOC120933211 | CD209 antigen-like protein C | pcadapt |
| LOC120933232 | uncharacterized LOC120933232 | pcadapt |
| LOC120933615 | uncharacterized protein LOC120933615 | pcadapt |
| LOC120933811 | uncharacterized LOC120933811 | bayenv2 (Climate PC3) |
| LOC120933833 | NACHT, LRR and PYD domains-containing protein 3-like | pcadapt |
| LOC120933847 | C-type lectin domain family 10 member A-like | pcadapt |
| LOC120934022 | vomeronasal type-2 receptor 26-like | pcadapt |
| LOC120935444 | vomeronasal type-2 receptor 26-like | bayenv2 (Climate PC3) |
| LOC120935633 | E1A-binding protein p400-like | bayenv2 (Climate PC3) |
| LOC120935661 | uncharacterized protein LOC120935661 | pRDA (Atrazine) |
| LOC120935833 | uncharacterized LOC120935833 | pcadapt |
| LOC120936310 | uncharacterized LOC120936310 | pRDA (Climate PC3) |
| LOC120936584 | uncharacterized LOC120936584 | RDA (Climate PC3) |
| LOC120936609 | carboxypeptidase B-like | pcadapt |
| LOC120937689 | sodium/calcium exchanger 1 | bayenv2 (Climate PC2) |
| LOC120937755 | WD repeat-containing and planar cell polarity effector protein fritz homolog | pcadapt |
| LOC120938372 | U5 spliceosomal RNA | pcadapt |
| LOC120938416 | small nucleolar RNA SNORA65 | pcadapt |
| LOC120938510 | uncharacterized gene | pcadapt |
| LOC120939638 | uncharacterized LOC120939638 | pcadapt |
| LOC120939715 | uncharacterized threonine-rich GPI-anchored glycoprotein PJ4664.02-like | bayenv2 (Climate PC3) |
| LOC120939943 | probable assembly chaperone of rpl4 | bayenv2 (Climate PC1) |
| LOC120940468 | sodium-dependent neutral amino acid transporter B(0)AT3-like | pcadapt |
| LOC120940533 | E3 SUMO-protein ligase ZBED1-like | bayenv2 (Climate PC3) |
| LOC120940612 | cadherin-10-like | pcadapt |
| LOC120940614 | uncharacterized LOC120940614 | bayenv2 (Climate PC3) |
| LOC120940829 | uncharacterized protein LOC120940829 | pcadapt |
| LOC120940884 | uncharacterized gene | pRDA (Atrazine) |
| LOC120941049 | cytochrome P450 7B1 | pRDA (Atrazine) |
| LOC120941054 | uncharacterized protein LOC120941054 | bayenv2 (Climate PC2) |
| LOC120941065 | uncharacterized gene | pcadapt |
| LOC120941494 | extensin-like | bayenv2 (Climate PC2) |
| LOC120941599 | flap endonuclease 1-like | bayenv2 (Climate PC2) |
| LOC120941686 | contactin-associated protein-like 4 | bayenv2 (Climate PC2) |
| LOC120941696 | spindle assembly abnormal protein 6 homolog | pcadapt |
| LOC120942167 | U1 spliceosomal RNA | bayenv2 (Climate PC2) |
| LOC120942646 | uncharacterized LOC120942646 | pcadapt |
| LOC120942669 | gamma-crystallin M2-like | pcadapt |
| LOC120942763 | taste receptor type 2 member 8-like | pcadapt |
| LOC120942810 | taste receptor type 2 member 39-like | pcadapt |
| LOC120943558 | uncharacterized protein LOC120943558 | bayenv2 (Climate PC3) |
| LOC120943613 | katanin-interacting protein | pRDA (2,4-D) |
| LOC120943720 | aldehyde oxidase 1-like | bayenv2 (Climate PC3) |
| LOC120943783 | carboxypeptidase O-like | bayenv2 (Climate PC2) |
| LOC120943822 | 1-phosphatidylinositol 3-phosphate 5-kinase-like | pcadapt |
| LOC120943823 | uncharacterized protein LOC120943823 | pRDA (Cropland) |
| LOC120944009 | neurabin-1-like | pcadapt |
| LOC120944316 | uncharacterized protein LOC120944316 | bayenv2 (Climate PC2) |
| LOC120944401 | protein ALP1-like | pcadapt |
| LOC120944410 | uncharacterized protein LOC120944410 | pcadapt |
| LOC120944551 | uncharacterized gene | pcadapt |
| LOC120945286 | cellular nucleic acid-binding protein | bayenv2 (Climate PC3) |
| LOC120945607 | pancreatic alpha-amylase-like | pcadapt |
| LOC120945747 | uncharacterized LOC120945747 | bayenv2 (Climate PC3) |
| LOC120946681 | C-type lectin domain family 2 member F-like | bayenv2 (Climate PC2) |
| LOC120946900 | transient receptor potential cation channel subfamily V member 6-like | pRDA (Climate PC1) |
| LRFN1 | leucine-rich repeat and fibronectin type III domain-containing protein 1 | RDA (Climate PC3) |
| LRRC20 | leucine-rich repeat-containing protein 20 | bayenv2 (Climate PC3) |
| MAPK6 | mitogen-activated protein kinase 6 | RDA (Climate PC2) |
| MBTPS2 | membrane-bound transcription factor site-2 protease | pRDA (2,4-D) |
| MCIDAS | multicilin | bayenv2 (Cropland) |
| ME3 | NADP-dependent malic enzyme, mitochondrial | pRDA (Climate PC1) |
| MED10 | mediator of RNA polymerase II transcription subunit 10 | bayenv2 (Climate PC2) |
| MELK | maternal embryonic leucine zipper kinase | bayenv2 (Nitrates) |
| MFAP3L | microfibrillar-associated protein 3-like | pRDA (Climate PC1) |
| MUCL3 | mucin-like protein 3 | bayenv2 (Climate PC1) |
| MYPN | myopalladin | pcadapt |
| NCOR1 | nuclear receptor corepressor 1 | bayenv2 (Climate PC1) |
| NDFIP2 | NEDD4 family-interacting protein 2 | bayenv2 (Climate PC3) |
| NEMF | nuclear export mediator factor NEMF | pcadapt |
| NFIB | nuclear factor 1 B-type | pcadapt |
| NKAIN2 | sodium/potassium-transporting ATPase subunit beta-1-interacting protein 2 | pRDA (Climate PC1) |
| NKX2-2 | homeobox protein Nkx-2.2 | pRDA (Climate PC1) |
| NPC1L1 | NPC1-like intracellular cholesterol transporter 1 | pRDA (Climate PC1) |
| NPFFR2 | neuropeptide FF receptor 2 | pRDA (Climate PC1) |
| NRAS | GTPase NRas | pcadapt |
| NTF3 | neurotrophin-3 | bayenv2 (2,4-D) |
| NTSR1 | neurotensin receptor type 1 | bayenv2 (Climate PC3) |
| OLFML3 | olfactomedin-like protein 3 | pRDA (Atrazine) |
| OLIG1 | oligodendrocyte transcription factor 1 | bayenv2 (Climate PC2) |
| OPN3 | opsin-3 | pRDA (Climate PC1) |
| PARP8 | protein mono-ADP-ribosyltransferase PARP8 | pcadapt |
| PC | pyruvate carboxylase, mitochondrial | RDA (Climate PC3) |
| PCGF2 | polycomb group RING finger protein 2 | bayenv2 (Climate PC2) |
| PCSK5 | proprotein convertase subtilisin/kexin type 5 | pcadapt |
| PDE1C | calcium/calmodulin-dependent 3',5'-cyclic nucleotide phosphodiesterase 1C | bayenv2 (Climate PC2) |
| PDE5A | cGMP-specific 3',5'-cyclic phosphodiesterase | pcadapt |
| PDPK1 | 3-phosphoinositide-dependent protein | pcadapt |
| PGM2 | phosphoglucomutase-2 | bayenv2 (Climate PC3) |
| PHETA1 | sesquipedalian-1 | bayenv2 (Climate PC3) |
| PIGN | GPI ethanolamine phosphate transferase 1 | pcadapt |
| PIK3C3 | phosphatidylinositol 3-kinase catalytic subunit type 3 | pcadapt |
| PIN1 | peptidyl-prolyl cis-trans isomerase NIMA-interacting 1 | pcadapt |
| PKD1 | polycystin-1 | pRDA (Climate PC3) |
| PLGRKT | plasminogen receptor (KT) | bayenv2 (Climate PC3) |
| PLXNA4 | plexin-A4 | RDA (Nitrates) |
| PNPLA4 | patatin-like phospholipase domain-containing protein 4 | bayenv2 (Climate PC3) |
| POLD1 | DNA polymerase delta catalytic subunit | pcadapt |
| POU4F3 | POU domain, class 4, transcription factor 3 | bayenv2 (Climate PC3) |
| PRCC | proline-rich protein PRCC | pcadapt |
| PRKD1 | serine/threonine-protein kinase D1 | bayenv2 (Climate PC1) |
| PSMG3 | proteasome assembly chaperone 3 | bayenv2 (Climate PC3) |
| PTBP2 | polypyrimidine tract-binding protein 2 | pRDA (Climate PC2) |
| PTPRC | receptor-type tyrosine-protein phosphatase C | pcadapt |
| QSOX2 | sulfhydryl oxidase 2 | bayenv2 (Cropland) |
| RAB4B | ras-related protein Rab-4B | pcadapt |
| RALYL | RNA-binding Raly-like protein | bayenv2 (Climate PC3) |
| RAPGEF5 | rap guanine nucleotide exchange factor 5 | pcadapt |
| RECK | reversion-inducing cysteine-rich protein with Kazal motifs | pcadapt |
| REV1 | DNA repair protein REV1 | bayenv2 (Climate PC2) |
| RFX1 | MHC class II regulatory factor RFX1 | pcadapt |
| RIOX1 | ribosomal oxygenase 1 | pRDA (Climate PC3) |
| RTN4R | reticulon-4 receptor | bayenv2 (Climate PC3) |
| SEC24A | protein transport protein Sec24A | pcadapt |
| SELENOF | selenoprotein F | pRDA (Climate PC1) |
| SERPINF2 | alpha-2-antiplasmin | bayenv2 (Atrazine) |
| SLC24A5 | sodium/potassium/calcium exchanger 5 | bayenv2 (Climate PC2) |
| SLC38A4 | sodium-coupled neutral amino acid transporter 4 | bayenv2 (Climate PC2) |
| SLC6A2 | sodium-dependent noradrenaline transporter | pcadapt |
| SLITRK4 | SLIT and NTRK-like protein 4 | bayenv2 (Climate PC3) |
| SMNDC1 | survival of motor neuron-related-splicing factor 30 | pRDA (Climate PC3) |
| SNU13 | NHP2-like protein 1 | pcadapt |
| SP4 | transcription factor Sp4 | pRDA (Atrazine) |
| SP8 | transcription factor Sp8 | pcadapt |
| SPAG16 | sperm-associated antigen 16 protein | pcadapt |
| SRRM3 | serine/arginine repetitive matrix protein 3 | pRDA (Climate PC1) |
| STT3B | dolichyl-diphosphooligosaccharide--protein glycosyltransferase subunit STT3B | bayenv2 (Climate PC3) |
| SYT17 | synaptotagmin-17 | pcadapt |
| SYTL1 | synaptotagmin-like protein 1 | pcadapt |
| TBC1D12 | TBC1 domain family member 12 | pcadapt |
| TCERG1L | transcription elongation regulator 1-like protein | pcadapt |
| TCTN2 | tectonic-2 | bayenv2 (Climate PC3) |
| TENM3 | teneurin-3 | pRDA (Atrazine) |
| THRA | thyroid hormone receptor alpha | bayenv2 (Climate PC2) |
| TMEM132B | transmembrane protein 132B | pcadapt |
| TMEM135 | transmembrane protein 135 | pcadapt |
| TMEM200A | transmembrane protein 200A | pRDA (Cropland) |
| TNRC18 | trinucleotide repeat-containing gene 18 protein | pcadapt |
| TNS3 | tensin-3 | bayenv2 (Climate PC3) |
| TRDN | triadin | pcadapt |
| TRNAQ-CUG | tRNA-Gln | bayenv2 (Climate PC1) |
| TRNAR-UCU-74 | tRNA-Arg | RDA (Climate PC1) |
| TRNAT-UGU-20 | tRNA-Thr | pcadapt |
| TRPV4 | transient receptor potential cation channel subfamily V member 4 | bayenv2 (Climate PC1) |
| TTC39C | tetratricopeptide repeat protein 39C | bayenv2 (Climate PC3) |
| UBR3 | E3 ubiquitin-protein ligase UBR3 | bayenv2 (Climate PC2) |
| WDR27 | WD repeat-containing protein 27 | pRDA (Climate PC1) |
| WNT2B | protein Wnt-2b | bayenv2 (Climate PC3) |
| WNT7B | protein Wnt-7b | pRDA (Climate PC1) |
| WWOX | WW domain-containing oxidoreductase | pcadapt |
| XKR8 | XK-related protein 8 | bayenv2 (Climate PC1) |
| XPO6 | exportin-6 | bayenv2 (Climate PC3) |
| ZC3H12D | probable ribonuclease ZC3H12D | pcadapt |
| ZC3H7B | zinc finger CCCH domain-containing protein 7B | bayenv2 (Climate PC3) |
| ZDHHC2 | palmitoyltransferase ZDHHC2 | bayenv2 (Climate PC2) |
| ZER1 | protein zer-1 homolog | bayenv2 (Climate PC2) |
| ZNF385B | zinc finger protein 385B | bayenv2 (Climate PC3) |
