# Supplementary figures and images for: Patterns of Local Adaptation in the Northern Leopard Frog (Rana pipiens) From a Region Undergoing Rapid Climate and Land‐Use Change
Source: Evol Appl. 2026 Jul 9;19(7):e70298. doi: 10.1111/eva.70298 (PMC13351322; doi:10.1111/eva.70298)

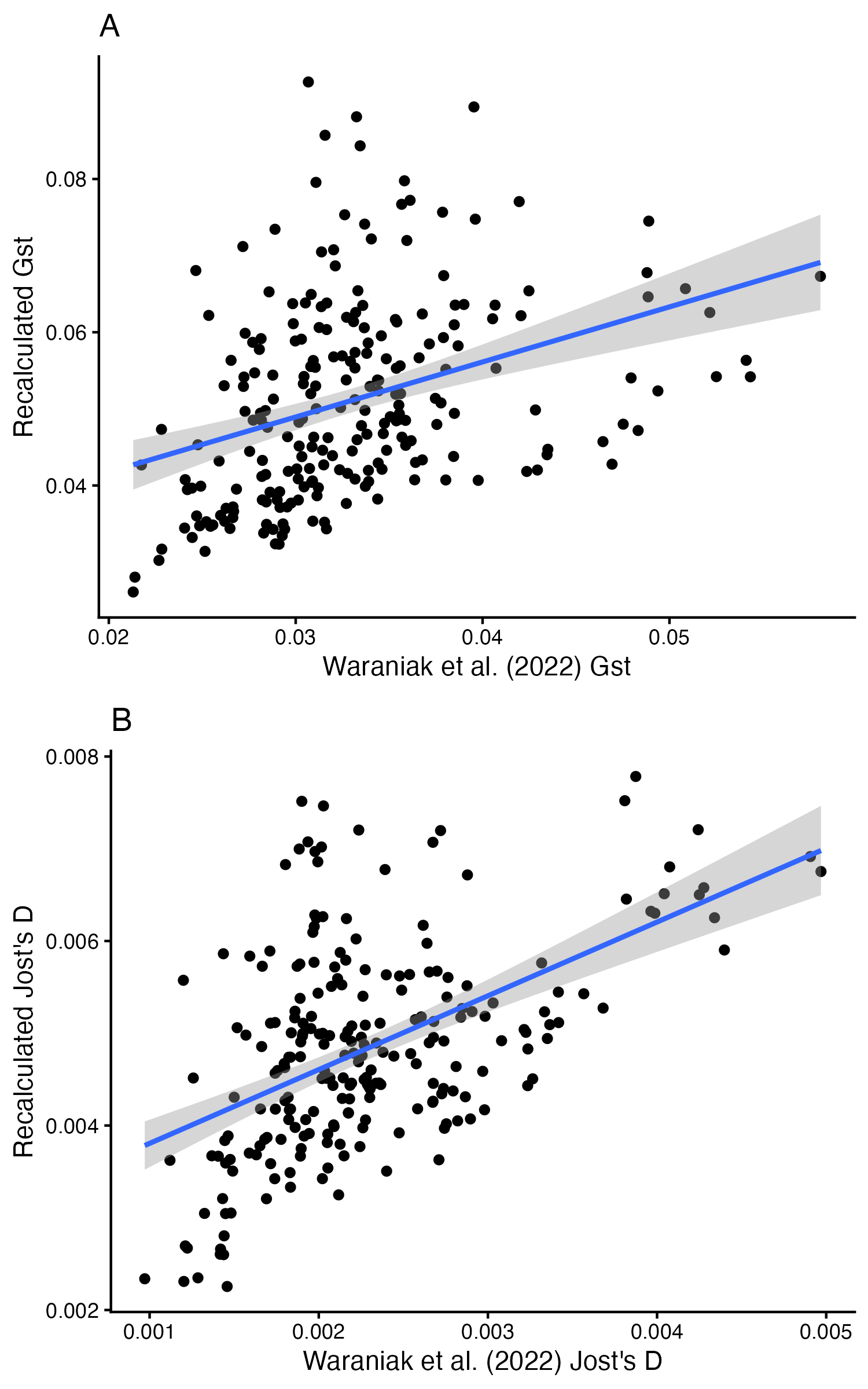

Supplement: Supplementary file 1 — Figure S1: Principal component of analysis of the 19 Worldclim bioclimatic variables at the 26 sample sites. Sample sites are represented by black dots, loadings of the bioclimatic variables along each axis are represented by red arrows and text. Figure S2: Metrics used to select the number of K clusters for the discriminant analysis of principal components on neutral population structure. BIC values for K = 2 and K = 4 are similar for the k‐means analysis. Figure S3: Comparison of genetic distances calculated from the dataset presented in Waraniak et al. (2022) to the genetic distances calculated from the current version of this dataset. Two genetic distances used to assess resistance surfaces were calculated, Nei's GST (A) and Jost's D (B). Table S1: Results of maximum likelihood population effects random effects models comparing the fits of resistance models using recalculated genetic distances to the genetic distances from Waraniak et al. (2022) and null models of geographic distance. Model fits were assessed using ∆BIC and marginal R 2 for two genetic distances, Nei's GST and Jost's D. Table S2: List of genes with gene symbol and gene name from the Rana temporaria reference genome that were aligned to loci identified as putatively under selection in Rana pipiens from our study area. Genes associate with SNPs identified by multiple genotype by association and outlier tests are listed first. [file EVA-19-e70298-s001.zip › Fig_S3.png]

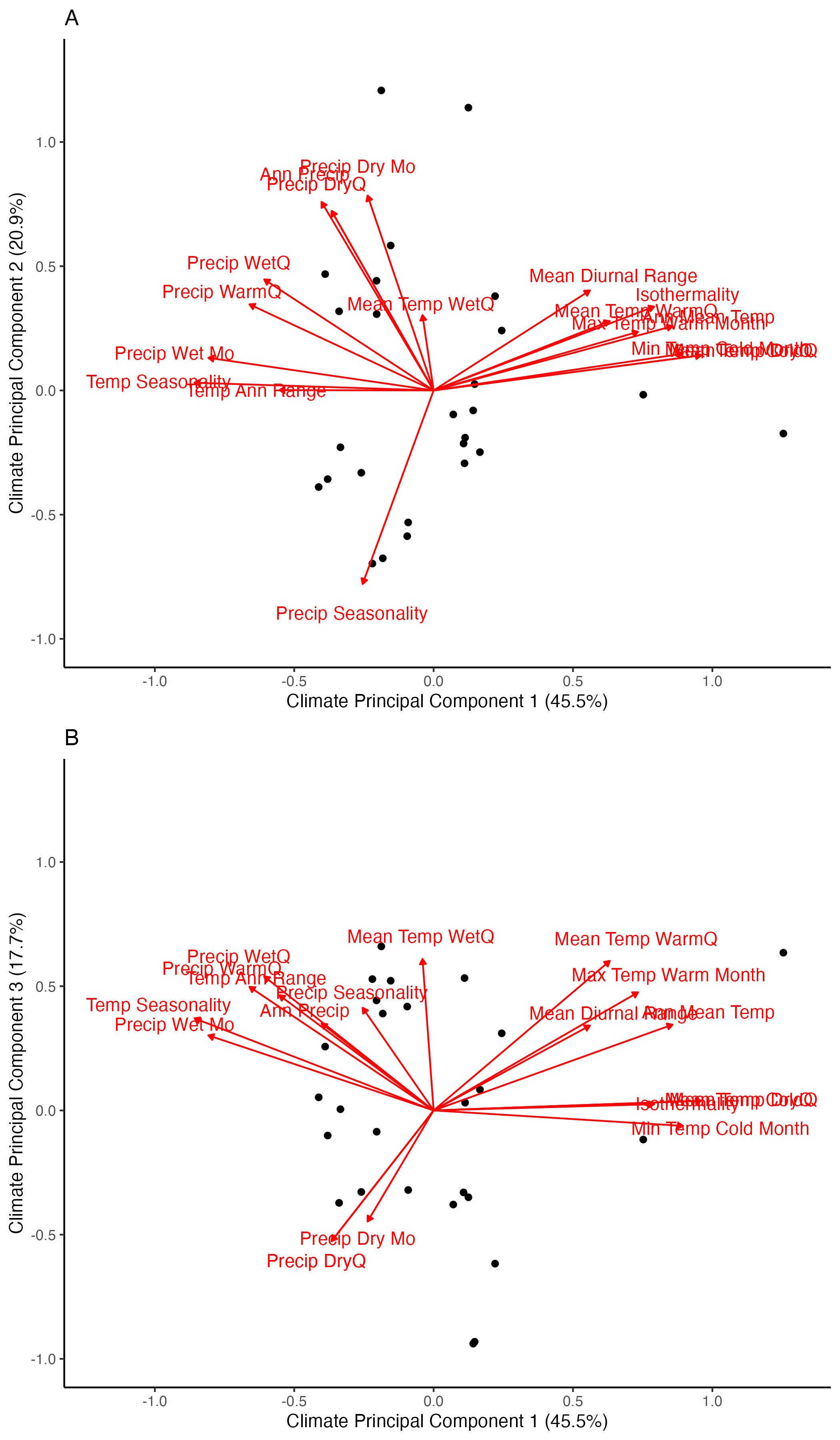

Supplement: Supplementary file 1 — Figure S1: Principal component of analysis of the 19 Worldclim bioclimatic variables at the 26 sample sites. Sample sites are represented by black dots, loadings of the bioclimatic variables along each axis are represented by red arrows and text. Figure S2: Metrics used to select the number of K clusters for the discriminant analysis of principal components on neutral population structure. BIC values for K = 2 and K = 4 are similar for the k‐means analysis. Figure S3: Comparison of genetic distances calculated from the dataset presented in Waraniak et al. (2022) to the genetic distances calculated from the current version of this dataset. Two genetic distances used to assess resistance surfaces were calculated, Nei's GST (A) and Jost's D (B). Table S1: Results of maximum likelihood population effects random effects models comparing the fits of resistance models using recalculated genetic distances to the genetic distances from Waraniak et al. (2022) and null models of geographic distance. Model fits were assessed using ∆BIC and marginal R 2 for two genetic distances, Nei's GST and Jost's D. Table S2: List of genes with gene symbol and gene name from the Rana temporaria reference genome that were aligned to loci identified as putatively under selection in Rana pipiens from our study area. Genes associate with SNPs identified by multiple genotype by association and outlier tests are listed first. [file EVA-19-e70298-s001.zip › FigS1.png]

**Value of BIC  
versus number of clusters**

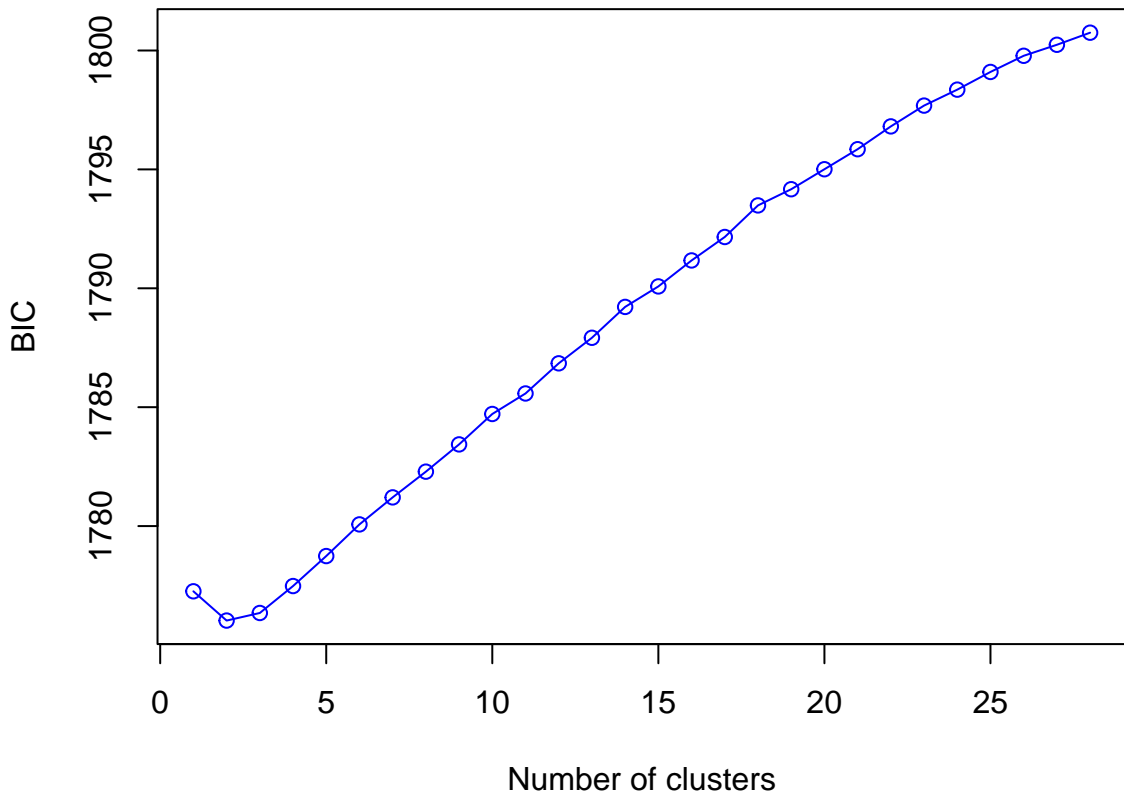

Supplement: Supplementary file 1 — Figure S1: Principal component of analysis of the 19 Worldclim bioclimatic variables at the 26 sample sites. Sample sites are represented by black dots, loadings of the bioclimatic variables along each axis are represented by red arrows and text. Figure S2: Metrics used to select the number of K clusters for the discriminant analysis of principal components on neutral population structure. BIC values for K = 2 and K = 4 are similar for the k‐means analysis. Figure S3: Comparison of genetic distances calculated from the dataset presented in Waraniak et al. (2022) to the genetic distances calculated from the current version of this dataset. Two genetic distances used to assess resistance surfaces were calculated, Nei's GST (A) and Jost's D (B). Table S1: Results of maximum likelihood population effects random effects models comparing the fits of resistance models using recalculated genetic distances to the genetic distances from Waraniak et al. (2022) and null models of geographic distance. Model fits were assessed using ∆BIC and marginal R 2 for two genetic distances, Nei's GST and Jost's D. Table S2: List of genes with gene symbol and gene name from the Rana temporaria reference genome that were aligned to loci identified as putatively under selection in Rana pipiens from our study area. Genes associate with SNPs identified by multiple genotype by association and outlier tests are listed first. [file EVA-19-e70298-s001.zip › Fig_S2.pdf]
